# Proteomics-Based Approach Identifies Altered ER Domain Properties by ALS-Linked VAPB Mutation

**DOI:** 10.1038/s41598-020-64517-z

**Published:** 2020-05-06

**Authors:** Tomoyuki Yamanaka, Risa Nishiyama, Tomomi Shimogori, Nobuyuki Nukina

**Affiliations:** 1 Laboratory of Structural Neuropathology, Doshisha University Graduate School of Brain Science, Kyoto, Japan; 2 Molecular Mechanisms of Brain Development, RIKEN Center for Brain Science, Saitama, 351-0198 Japan

**Keywords:** Motor neuron disease, Endoplasmic reticulum, Protein aggregation, Proteomic analysis

## Abstract

An ER transmembrane protein, vesicle-associated membrane protein-associated protein B (VAPB), binds to several organelle-resident membrane proteins to mediate ER-organelle tethering. Mutation in amyotrophic lateral sclerosis (ALS) induces protein misfolding and aggregation, leading to ER disorganization. Gain or loss of function is suggested for VAPB mutation, however comprehensive study focusing on VAPB-ER domain has yet been performed. We here conducted proteomic characterization of the ER containing VAPB and its ALS-linked P56S mutant. For this purpose, we first optimized the proteomics of different ER domains immuno-isolated from cultured cells, and identified ER sheet- and tubule-specific proteomes. By using these as references, we found that VAPB-ER proteome had intermediate ER domain properties but its tubular property was specifically decreased by its mutation. Biochemical, immunofluorescence and proximity ligation assays suggested this was mediated by delocalization of VAPB from ER tubules. The VAPB-ER proteomics further suggested reduced incorporation of multiple proteins located in different organelles, which was confirmed by proximity ligation assay. Taken together, our proteomics-based approach indicates altered ER domain properties and impaired ER-organelle tethering by VAPB mutation.

## Introduction

The endoplasmic reticulum (ER) is a continuous membrane organelle dispersing throughout the cells. It plays key roles in protein synthesis/transport, lipid/sterol synthesis, calcium storage and metabolism. These multiple cellular functions are differentially regulated by morphologically distinct two ER domains, sheets and tubules. The sheets exist as cisternae and tend to be studded with ribosomes, whereas the tubules form tubular networks and are largely devoid of ribosomes^1–4^. Different sets of ER-resident membrane proteins are involved in the structural organization of these distinct ER domains; integral transmembrane proteins such as Climp-63 for sheets, and hairpin transmembrane proteins such as DP1 and reticulons for tubules^1,2,5,6^. In addition, ER is interacting with multiple membranous organelles such as mitochondria and plasma membranes and meditate inter-organelle transfer of ions and lipids. These interactions are mediated by several tethering transmembrane proteins^7,8^. However, the relationship between the ER domains and organelle interactions, as well as the actual localization of the tethering proteins in ER domains, has not been fully characterized.

Vesicle-associated membrane protein-associated protein B (VAPB) is an ER integral transmembrane protein involved in ER-organelle tethering^9,10^. VAPB and its closely related protein VAPA are composed of N-terminal major sperm protein (MSP), coiled coil and transmembrane domains. The MSP domain facing at cytoplasm binds to FFAT (two phenylalanines in an acidic tract) motif to mediate interactions with multiple proteins located in various cellular compartments including mitochondria^11,12^, Golgi network^13,14^, peroxisome^15,16^, endosome^17^ and plasma membrane^18,19^. These observations highlight a pivotal role of VAPB as a molecular bridge at inter-organelle contact sites.

VAPB is shown to be mutated in a familial motor neuron disease, amyotrophic lateral sclerosis (ALS) type 8^20^. The VAPB gene product containing P56S mutation is misfolded and aggregated on ER membrane, leading to ER disorganization characterized by formation of clustered ER inclusions^21–25^. Notably, the VAPB mutant is shown to sequester several VAPB interactors including PTPIP51 and further cluster organelles around the VAPB aggregates^12,14,16^, suggesting gain of toxic tethering function involved in ALS pathogenesis. However, other observations suggest loss of FFAT interaction by VAPB mutation^22^. Further, recent knockout or knockin studies suggest the involvement of loss of function by VAPB mutant in neuronal dysfunction^26–28^. Reduced expression of human VAPB is further reported in motor neurons of sporadic ALS cases^22,29^. Thus, in addition to the gain of toxic function, loss of function is also suggested and actual mechanism underlying disease progression by the mutation still remains uncertain.

In this study, we conducted proteomic characterization of the ER domains containing VAPB wild type and its P56S mutant. For this purpose, we first identified ER sheet- and tubule-specific proteomes in cultured cells. By using these proteomes as references, we found decreased tubular property of VAPB-containing ER by ALS-linked mutation. Our analysis further suggests reduced VAPB interaction with the proteins in other organelles, and supports the impairment of ER-organelle tethering by the mutation.

## Results

### Exclusive distribution of EGFP-tagged ER sheet and tubule proteins in N2a cells

By using Flp-in system, we generated N2a mouse neuroblastoma cell lines expressing EGFP- or mRFP-tagged ER membrane proteins enriched in sheets (Climp63) and tubules (DP1)^30–32^ (Fig. S1A). Staining of the cell lines with α-tubulin revealed that EGFP-Climp63 was lesser in neurites, whereas EGFP-DP1 was highly detected there (Fig. S1B). The difference was also observed in their localizations in cell body; EGFP-DP1 showed focused distribution at perinucleus whereas EGFP-Climp63 was distributed in cell bodies except of the EGFP-DP1-positive regions (Fig. S1C). We confirmed that their distributions were very similar to endogenous Climp63 and another ER tubule-enriched protein Reticulon 4 (Rtn4; isoform A) (Fig. S1A,D,E). The perinuclear Rtn4 accumulation was found around γ-tubulin-positive centrosome (Fig. S1E). Taken together, these data indicate that in N2a cells ER tubules are rich in neurites and around centrosomes whereas ER sheets are dominantly distributed in cell bodies except of the ER tubules-enriched regions, and this exclusive distribution was preserved for exogenous EGFP-tagged Climp63 and DP1.

**Figure 1:**
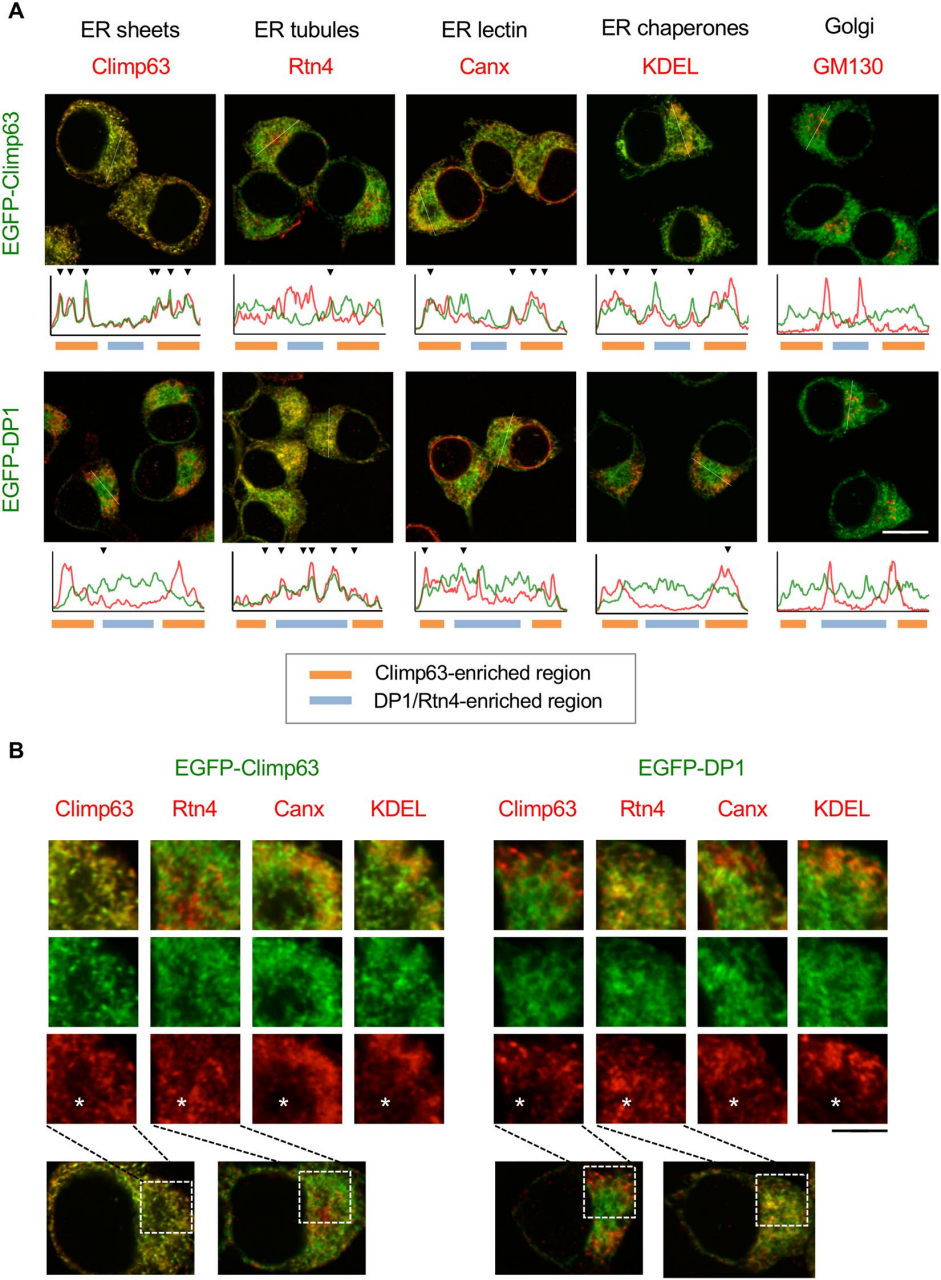
Differential distribution of EGFP-tagged sheet/tubule proteins in N2a cells. (**A**) N2a cell line expressing EGFP-Climp63 (upper panels) or EGFP-DP1 (lower panels) (green) was stained with indicated marker proteins for ER and Golgi (red). Fluorescence intensities of the regions indicated by white lines are plotted on the bottom of the panels, and well co-distributions between green (EGFP) and red (markers) are indicated by arrowheads. Regions enriched for Climp63 and DP1/Rtn4 are also indicated in bottom lines with different colors. (**B**) Higher magnification of the cell body regions (boxes in the lower panels). Asterisks indicate the DP1/Rtn4-eiriched regions. Note the almost exclusive localizations of EGFP-Climp63 with ER tubule Rtn4, and EGFP-DP1 with sheet Climp63. Canx and KDEL were relatively well co-distributed with EGFP-Climp63 rather than EGFP-DP1, whereas GM130 was not co-distributed with them. Scale bars are 10 μm (**A**) and 4 μm (**B**).

Because at least in N2a cells the cell body region is useful to analyze the specific distributions of ER sheet/tubule proteins at the same confocal plane, we compared the distribution of EGFP tagged proteins with several markers in this region (Fig. 1A,B). As expected, EGFP-Climp63 showed less co-distribution with endogenous Rtn4, but partly with an ER lectin, calnexin (Canx), and an ER luminal marker, KDEL. In contrast, EGFP-DP1 showed well co-distribution with Rtn4, partly with Canx and KDEL but lesser with endogenous Climp63. Finally, all of EGFP proteins were not co-localized with a Golgi marker GM130. These data also support the idea of exclusive distributions of EGFP-tagged sheet and tubule proteins in ER.

### Immuno-isolation of different ER domains containing Climp63 and DP1

**Figure 2:**
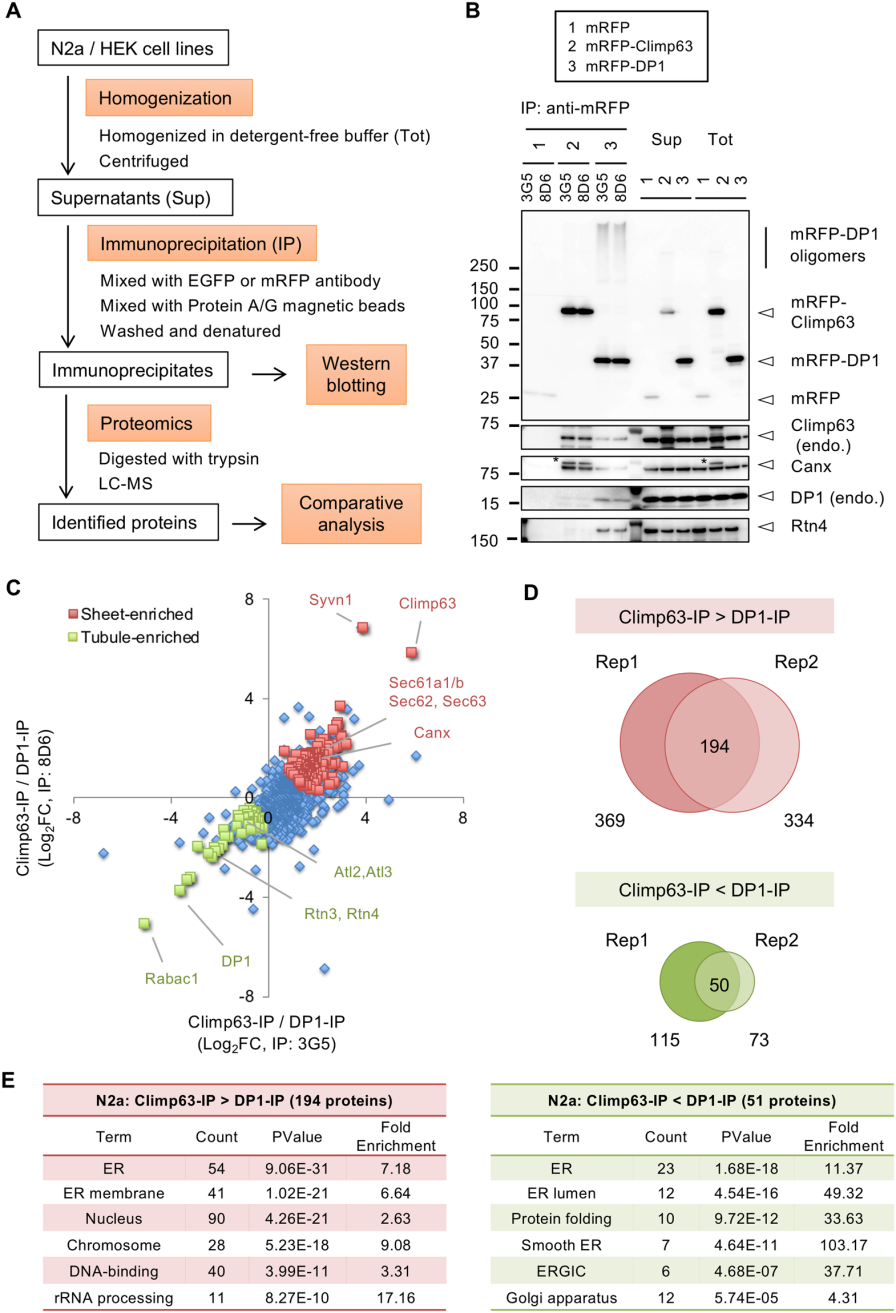
Proteomic analysis of the immunoprecipitates for mRFP-Climp63 and mRFP-DP1 isolated from N2a cells in a detergent-free condition. (**A**) Schematic procedure of immuno-isolation and proteomics of different ER domains. The cell lines were homogenized in detergent-free sucrose buffer and subjected to immunoprecipitation (IP), followed by Western blotting or LC-MS. (**B**) Western blotting of the immunoprecipitates by anti-mRFP antibodies (3G8 and 8D6) from N2a cell lines expressing mRFP, mRFP-Climp63 and mRFP -DP1. Precipitated proteins were analyzed by antibodies for indicated proteins (*non-specific bands). (**C-E**) Obtained immunoprecipitates were digested with trypsin and analyzed by LC-MS. Proteome Discoverer version 2.2 was used to identify the proteins and quantify their abundancies (label free quantification). After normalization with the amount of precipitated mRFP-tagged proteins, protein abundancies were compared between the two IP sets and log2 values of the fold changes (FCs; mRFP-Climp63-IP/mRFP-DP1-IP) were calculated. (**C**) Scatter plot of log_2_FCs for two IP sets (3G5 and 8D6). Proteins enriched by mRFP-Climp63-IP (red) or mRFP-DP1-IP (green) in two biological replicates (**D**) were colorized and known sheets and tubule proteins are indicated. (**D**) Identified proteins enriched in mRFP-Climp63-IP (upper diagram) or in mRFP-DP1-IP (lower diagram) in two biological replicates (rep 1 and 2); total 194 and 50 proteins were identified in both replicates, respectively. (**E**) Identified proteins were processed for functional annotation analysis. Proteins related to nucleus were relatively abundant in the mRFP-Climp63-IP, whereas proteins related to ER lumen, Golgi apparatus and chaperone were abundant in the mRFP-DP1-IP.

To perform proteomic characterization of ER sheets and tubules, we tried to isolate these domains by immunoprecipitation (IP) (Fig. 2A). To this end, we homogenized N2a cells expressing mRFP-Climp63 and mRFP-DP1 with detergent-free buffer to preserve membranous structures, followed by brief sonication for efficient segregation of the ER domains. For IP we used two different antibodies for mRFP (3G5 and 8D6) or EGFP (598 and D153) to avoid incorporation of non-specific proteins, and used Pierce magnetic beads for efficient IP of relatively larger membranous structures. For negative control, the cells expressing mRFP/EGFP-tag only were used.

Western blotting of the IP samples revealed efficient precipitation of mRFP-Climp63 and mRFP-DP1 by both mRFP antibodies from N2a cells (Fig. 2B). Endogenous Climp63 and Canx were well co-precipitated with mRFP-Climp63, whereas endogenous DP1 and Rtn4 were not. In contrast, endogenous DP1 and Rtn4 were well precipitated with mRFP-DP1 whereas endogenous Climp63 and Canx were lesser. These co-precipitations were unobserved in control IPs. These exclusive precipitation of ER proteins were reproduced in another set of IPs from N2a cells (Fig. S2A), and mostly correspond to the data of immuno-staining (Fig. 1). Our data are totally consistent with previous reports; Climp63 and Canx are abundant in rough microsome (rough ER sheets) fraction prepared from dog pancreas^31^, whereas reticulons are enriched in isolated tubular microsome (ER tubules) containing Yop1p (a yeast DP1 homologue) from yeasts^33^. We thus suggest successful isolation of different ER domains, the sheets and tubules, by our IP method.

### Proteomic characterization of the Climp63 and DP1-containing ER domains

To identify the proteins incorporated in different ER domains, we performed proteomic analysis of the IP samples obtained above (Fig. 2A). The proteins were digested with trypsin and processed for liquid chromatography-mass spectrometry (LC-MS) analysis. Based on the peptide spectra and abundancies, protein identities and its abundancies (label free quantification) were obtained by Proteome Discoverer software 2.2^34^. After normalization with the amount of precipitated mRFP-tagged proteins, protein abundancies were compared between the two IP sets and log2 values of the fold changes (FCs; mRFP-Climp63-IP/mRFP-DP1-IP) were calculated. Scatter plot of the log_2_FCs for two IP sets (3G5 and 8D6) clearly revealed positive scores (enriched in Climp63-IP) for known sheet proteins including Sec61/62/63 in addition to Climp63, and negative scores (enriched in DP1-IP) for tubule proteins including reticulons (Rtn2/3) and atlastins (Atl2/3) in addition to DP1 (Fig. 2C). Canx was also confirmed to be enriched in Climp63-IP by the proteomics (Fig. 2C). Differential enrichment of these proteins was reproduced by another set of IP-proteomics (Fig. S2B). By comparing these replicates, we finally identified 194 and 50 proteins enriched in Climp63-IP and DP1-IP, respectively (Fig. 2C,D, Table S1, 2). Functional annotation analysis revealed relative enrichment of ER membrane, nuclear and RNA-related proteins in the Climp63-IP; and enrichment of ER lumen and Golgi proteins in the DP1-IP (Fig. 2E), suggesting that proteins with different locations/functions were abundant in the ER sheets and tubules.

We also performed IP-proteomics from HEK293 cells expressing EGFP-tagged Climp63 or DP1. Similar to N2a cells, Western blot and proteomic analyses revealed enrichment of known ER sheet and tubule proteins in the EGFP-Climp63-IP and EGFP-DP1-IP from HEK293 cells, respectively (Fig. S3A,B, Tables S3, 4). Again, ER membrane, nuclear and RNA-related proteins were relatively enriched in the Climp63-IP from HEK293 cells whereas ER lumen and Golgi proteins were enriched in the DP1-IP (Fig. S3C). Also, identified sheets and tubules-enriched proteins in N2a cells (Fig. 2D) also tended to be enriched in Climp63-IP and DP1-IP from HEK cells, respectively (Fig. S3D), suggesting preservation of ER domain-specific protein compositions among the different cell types.

We then checked distributions of the identified proteins in cultured cells. Basically, central cell body region is rich in ER sheets whereas cell peripheral region mainly contains ER tubules^1,30,31^. We obtained immunofluorescence cell images for identified proteins from the Human Protein Atlas database^35,36^, and quantified fluorescence signals from center to periphery (regions 1~4) (Figs. S4, S5). We observed intensities high in center (regions 1~2) for known ER sheet proteins (Fig. S4A,B,D), whereas they were relatively remained to periphery (regions 3~4) for known ER tubule proteins (Fig. S5A,B,D). Similar tendencies were observed for the identified ER membrane proteins enriched in Climp63-IP (Fig. S4C,E) or in DP1-IP (Fig. S5C,E) although some variations were observed. These cell image analyses implied overall difference in cellular distribution of the identified proteins, however further detailed analyses will be necessary to validate their domain-specific distributions.

### Proteomic characterization of the ER domains containing VAPB and its ALS-linked mutant

To characterize the ER domains positive for VAPB and its alteration by VAPB mutation, we performed proteomics of the isolated ERs containing VAPB and its P56S mutant. To this end, we generated N2a cell lines expressing VAPB-wt and VAPB-P56S tagged with EGFP at their N-terminus, and performed immunoprecipitation using two different EGFP antibodies (598 and D153). The precipitates were processed for by LC-MS, and precipitated proteins were identified and quantified as we did above.

**Figure 3:**
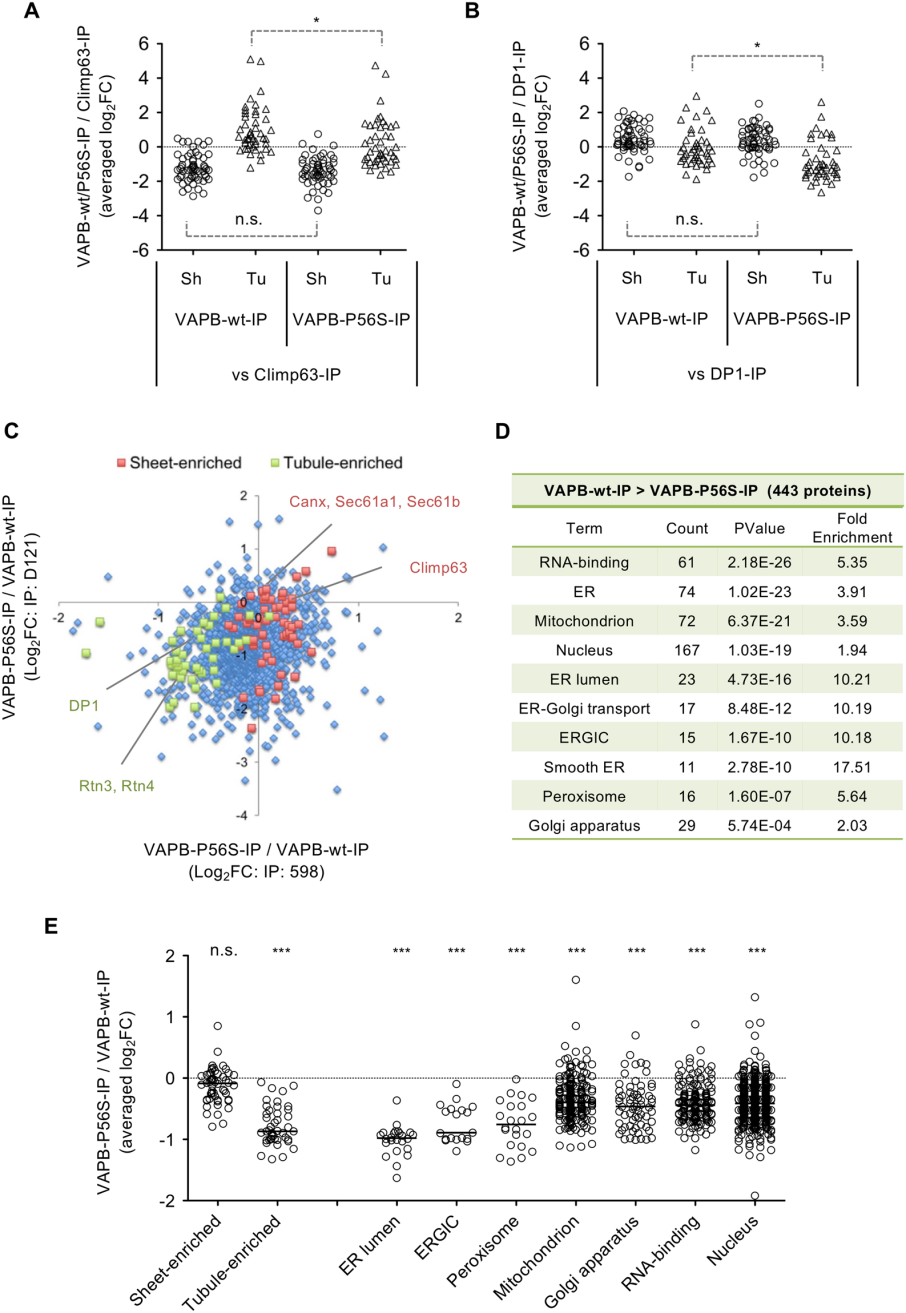
Proteomic analysis of the immunoprecipitates for EGFP-VAPB-wt and its P56S mutant from N2a cells. N2a cell lines expressing EGFP-VAPB-wt and EGFP-VAPB-P56S were homogenized in detergent-free sucrose buffer and subjected to immunoprecipitation using two anti-EGFP antibodies (598 and D153). Immunoprecipitates were digested with trypsin and analyzed by LC-MS. Proteome Discoverer version 2.2 was used to identify the proteins and quantify their abundancies (label free quantification). (**A**,**B**) Comparison of the protein abundancies in EGFP-VAPB-IP (wt or P56S) with those for mRFP-Climp63-IP (**A**) or mRFP-DP1-IP (**B**). Averaged log_2_ values of the fold changes (FCs) were plotted. Only the data for proteins enriched in ER sheets (Sh) and tubules (Tu) (Table S1, S2) were shown. The data were statistically analyzed by one-way ANOVA followed by Tukey post-test (*significant, n.s.; not significant). (**C**) Protein abundancies were compared between the EGFP-VAPB-wt-IP and EGFP-VAPB-P56S-IP, and Log_2_FCs (EGFP-VAPB-P56S-IP/EGFP-VAPB-wt-IP) were plotted. Labeling of the proteins enriched in ER sheets and tubules (Table S1, S2) revealed preferential reduction of the tubule proteins in the EGFP-VAPB-P56S-IP. (**D**) Identified 443 proteins reduced in EGFP-VAPB-P56S-IP were processed for functional annotation analysis. Proteins related to nucleus and ER lumen, mitochondria, Golgi apparatus and peroxisome were relatively enriched. (**E**) Averaged log_2_FCs of the ER sheets (Sh) and tubules (Tu)-enriched proteins, as well as those for proteins related to indicated cellular locations/functions were plotted. The data were statistically analyzed by one-way ANOVA followed by Tukey post-test (***significant, n.s.; not significant).

Comparison with Climp63-IP proteome revealed relative increase in tubule-enriched proteins in VAPB-wt-IP (Fig. 3A). In contrast, comparison with DP1-IP proteome revealed slight increase in sheet-enriched proteins (Fig. 3B). These suggest that both tubule and sheet proteins were relatively contained in the VAPB-wt-IP proteome. Notably, the tubule proteins were slightly but significantly reduced in VAPB-P56S-IP proteome (Fig. 3A,B). To clarify this, we directly compared the proteome of VAPB-P56S-IP with that of VAPB-wt-IP (Table S5), and found specific reduction of ER tubule but not sheet proteins in the VAPB-P56S-IP (Fig. 3C,E). Functional annotation analysis also showed reduced incorporation of the proteins with known ER tubule properties (Fig. 2E), such as ER lumen, ERGIC, smooth ER and Golgi apparatus in the VAPB-P56S-IP (Fig. 3D,E). In addition, we further found the reduced incorporation of mitochondrial and peroxisomal proteins in the VAPB-P56S-IP (Fig. 3D,E). Reduced incorporation of ER tubule properties and some of Golgi, mitochondrial and peroxisomal proteins were reproduced by another set of IP-proteomics (Fig. S6, Table S6). Taken together, these data suggest that VAPB-IP proteome has intermediate ER domain properties, but ER tubule property was specifically decreased by P56S mutation. The mutation may also affect the interaction of VAPB-containing ERs with other organelles.

### Delocalization of VAPB mutant from ER tubules

**Figure 4:**
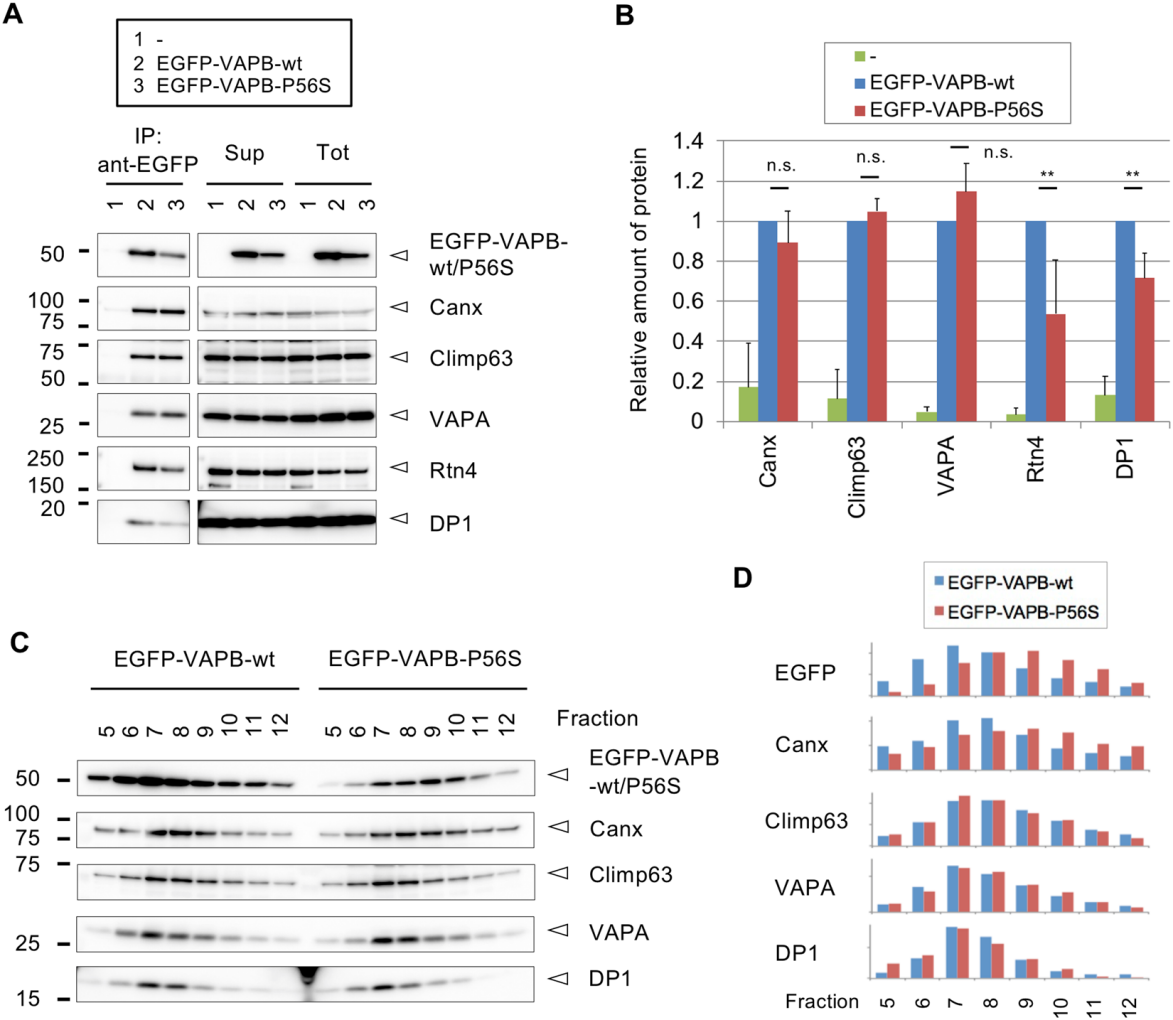
IP-Western blot and Percoll fractionation analyses for N2a cells expressing EGFP-VAPB-wt and its P56S mutant. (**A**) N2a cells or those expressing EGFP-VAPB-wt and EGFP-VAPB-P56S homogenized in detergent-free sucrose buffer (Tot) were centrifuged (Sup) and subjected to immunoprecipitation using anti-EGFP antibody (598). Immunoprecipitates were analyzed by Western blotting using antibodies for indicated proteins. (**B**) Quantification of the co-precipitated proteins in A. Values are means + SD of three or four independent experiments and statistically analyzed by one-way ANOVA followed by Tukey post-test (**significant, n.s.; not significant). (**C**) The N2a cell lines for EGFP-VAPB-wt and EGFP-VAPB-P56S were homogenized in detergent-free sucrose buffer and subjected to ultracentrifucation in 30% Percoll/sucrose buffer. Twenty fractions were collected from the top, and fractions 5–12 were analyzed by Western blotting for indicated proteins. (**D**) Quantification of the fractionated proteins in C. Values are means of two independent experiments.

To confirm the proteomic data described above, we performed Western blot analysis of the immunoprecipitates for EGFP-VAPB-wt and its P56S mutant. We observed co-precipitation of ER-sheet (Climp63 and Canx) and tubule (Rtn4 and DP1) proteins with EGFP-VAPB-wt by Western blotting (Fig. 4A), supporting the intermediate domain properties of the EGFP-VAPB-wt-IP proteome. VAPB-related protein VAPA was also co-precipitated, consistently with their close co-distribution in the cells (Fig. S7). Even though the amount of EGFP-VAPB-P56S was relatively low possibly because of its degradation through ubiquitin-proteasome system^21^, Canx, Climp63 and VAPA were similarly co-precipitated (Fig. 4A,B). In contrast, co-precipitated Rtn4 and DP1 was significantly decreased in the EGFP-VAPB-P56S-IP (Fig. 4A,B). These are highly compatible with the data of IP-proteomics (Fig. 3), that is, incorporation of ER tubule proteins was specifically reduced in the EGFP-VAPB-P56S-IP, however less mutant protein in the IP could be involved in this reduction. To clarify the reduced interaction of VAPB mutant with ER tubules, we further performed following experiments.

We first performed density gradient fractionation of cell homogenates in a colloidal silica Percoll^37,38^, and found co-fractionation of EGFP-VAPB-wt with Canx, Climp63, VAPA and DP1, among which DP1 distribution was relatively restricted to the peak fractions (Fig. 4C,D). In contrast, EGFP-VAPB-P56S as well as Canx was found to be shifted to later fractions where DP1 was lowly detected (Fig. 4C,D). Shift to later fractions of the mutant protein was reproduced by another set of fractionations (Fig. S8). These data also support the notion of reduced incorporation of ER tubule protein in the ER domain containing EGFP-VAPB-P56S.

**Figure 5:**
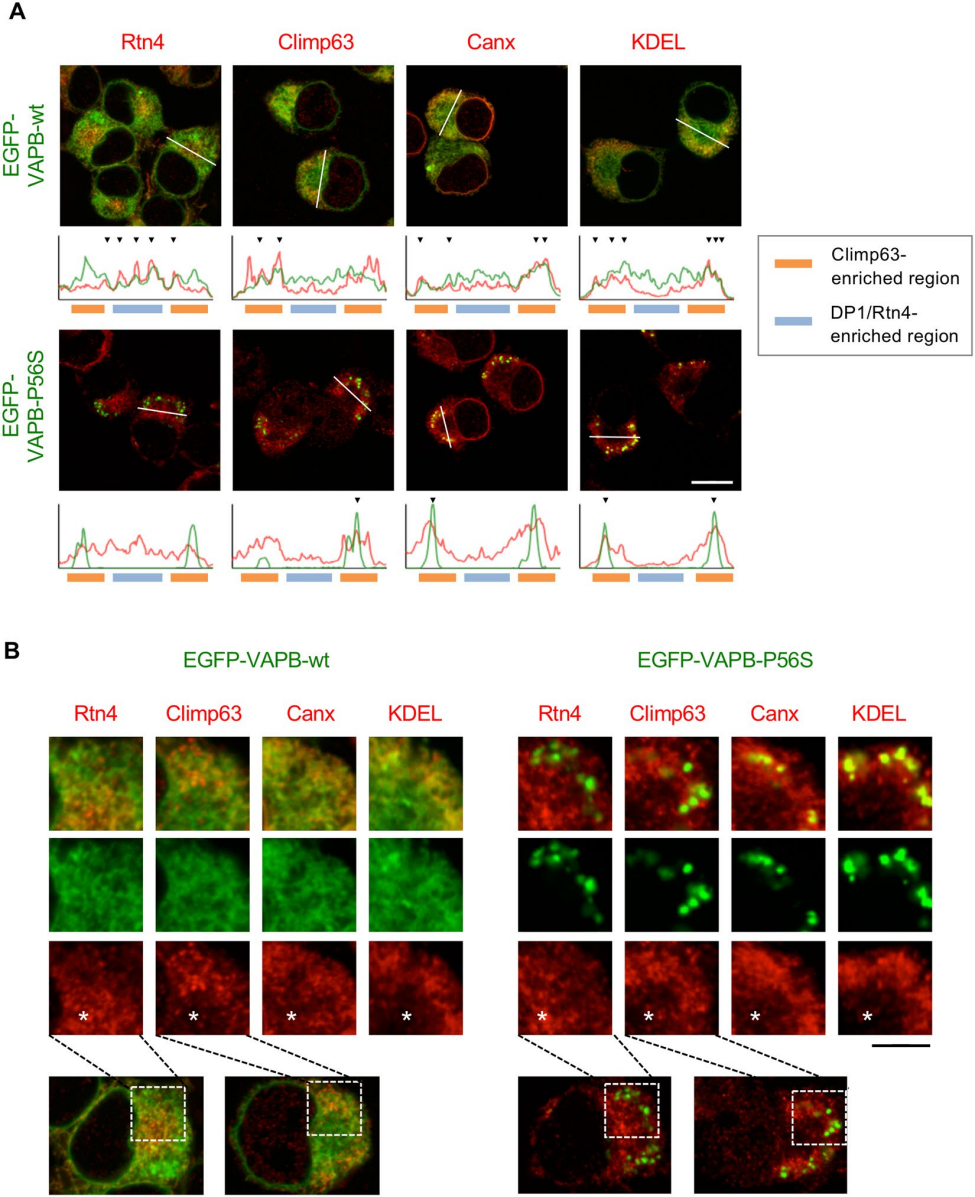
Immunofluorescence analyses for N2a cells expressing EGFP-VAPB-wt and its P56S mutant. (**A**) N2a cell line expressing EGFP-VAPB-wt (upper panels) or EGFP-VAPB-P56S (lower panels) (green) was stained with indicated ER proteins (red). Fluorescence intensities of the regions indicated by white lines are plotted on the bottom of the panels, and well co-distributions between the green and red signals are indicated by arrowheads. Regions enriched for Climp63 and DP1/Rtn4 are also indicated in bottom lines with different colors. (**B**) Higher magnification of the cell body regions (boxes in the lower panels). Asterisks indicate the DP1/Rtn4-eiriched regions. Note the uniform distribution of EGFP-VAPB-wt in colocalization with peripheral sheet-enriched Climp63, Canx and KDEL, as well as perinuclear tubule Rtn4, whereas aggregated EGFP-VAPB-P56S was excluded from the region positive for perinuclear Rtn4. Scale bars are 10 μm (**A**) and 4 μm (**B**).

Next, we examined cellular distribution of EGFP-VAPB in N2a cells. EGFP-VAPB-wt showed uniform ER localization and well co-distributed with endogenous VAPA (Fig. S7), supporting the physiological distribution of EGFP-VAPB-wt in N2a cells. In contrast, EGFP-VAPB-P56S showed aggregated localization where partial accumulation of VAPA was detected (Fig. S7), suggesting sequestration of VAPA by mutant VAPB aggregates^22,39^. Co-distribution with all of tested ER proteins, Rtn4, Climp63, Canx and KDEL (Fig. 5A,B) suggests ubiquitous distribution of EGFP-VAPB-wt in both ER sheet and tubules in N2a cells. Interestingly, EGFP-VAPB-P56S was mostly excluded from the Rtn4-positive perinuclear regions and aggregated in the regions enriched in Climp63, Canx and KDEL (Fig. 5A). Detailed analysis suggests although Climp63 showed close localization to EGFP-VAPB-P56S aggregates it did not accumulate to them whereas partial co-accumulation was observed for Canx and KDEL (Fig. 5B).

**Figure 6:**
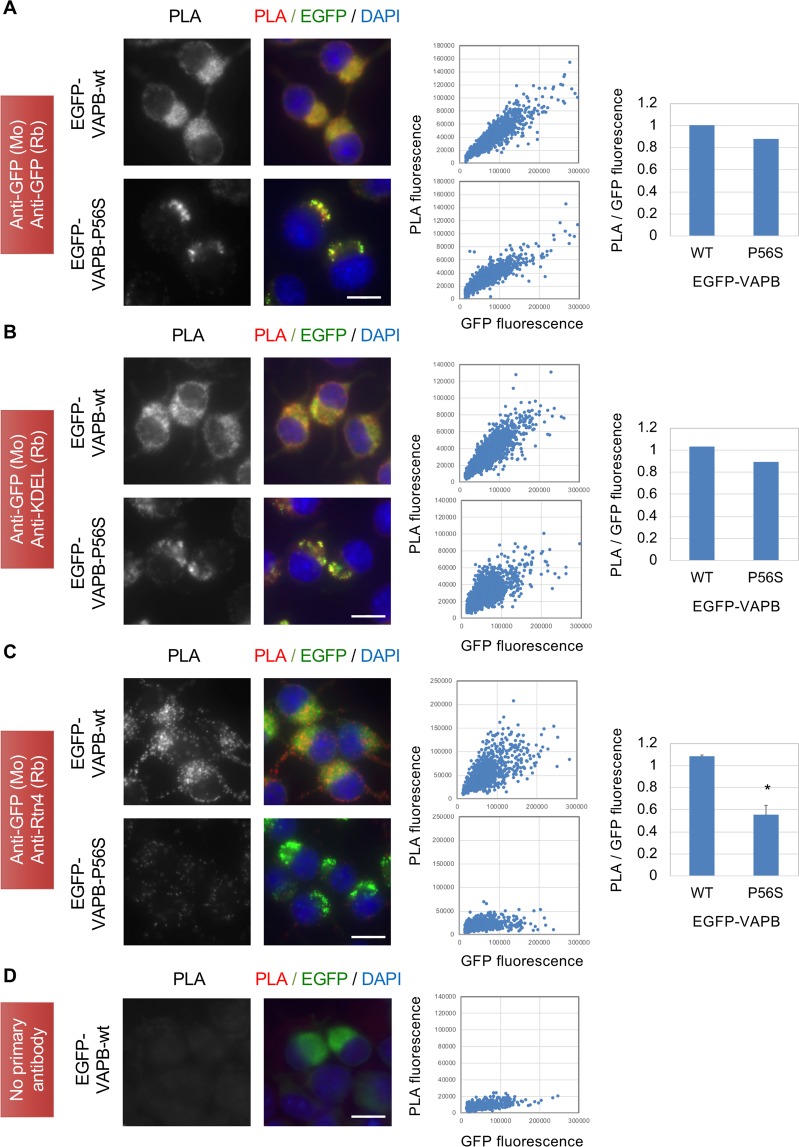
Proximity analysis of EGFP-VAPB to ER sheet/tubule proteins in N2a cells. N2a cell lines expressing EGFP-VAPB-wt or EGFP-VAPB-P56S were subjected to PLA using antibody for EGFP (mouse) together with those for EGFP (rabbit) (**A**), KDEL (**B**) or Rtn4 (**C**). (**D**) No primary antibodies were used. PLA signals and merged views with EGFP and DAPI are shown (left panels, scale bars are 10 μm). Fluorescence intensities for PLA and EGFP in each cell were quantified by cell image analyzer and blotted (middle panels). After normalization with their median values, ratios of PLA to EGFP intensities were calculated, and means of two (**A**,**B**) or three (+ SD) (**C**) independent experiments (*P < 0.05, Welch’s t test) are shown (right panels).

Finally, we performed proximity ligation assay (PLA)^40,41^ to confirm the disturbed ER tubule-localization of VAPB mutant. Because ER luminal thickness in N2a cells is ~60 nm that is two third of the theoretical maximum distance of the PLA antibodies for ligation (40 nm), we expected co-distribution of two proteins on the same ER domain could induce PLA signal to some extent. We first used two GFP antibodies derived from rabbit or mouse as a positive control and observed clear PLA signals for both EGFP-VAPB-wt and EGFP-VAPB-P56S in the regions positive for EGFP fluorescence (Fig. 6A). We next used GFP and KDEL antibodies and also found PLA signals for both VAPB proteins (Fig. 6B) in the regions those are highly compatible with the co-stained regions observed by immunofluorescence microscopy (Fig. 5). In contrast, when antibodies for GFP and Rtn4 were used, PLA signals observed in perinuclear and nuritic regions of EGFP-VAPB-wt-expressing cells were drastically reduced in the cells expressing EGFP-VAPB-P56S (Fig. 6C, 55% to the EGFP-VAPB-wt). These PLA signals are specific because no distinct signals were detected if no primary antibody was used (Fig. 6D). We also validated specificities of the antibodies used in these assays by Western blotting (Fig. S9). Taken together, these data indicate that VAPB is contained in both ER sheet and tubule domains but is delocalized from the tubules upon P56S mutation and accumulated around the ER sheets.

### Reduced proximity of VAPB with the proteins located in other organelles by P56S mutation

**Figure 7:**
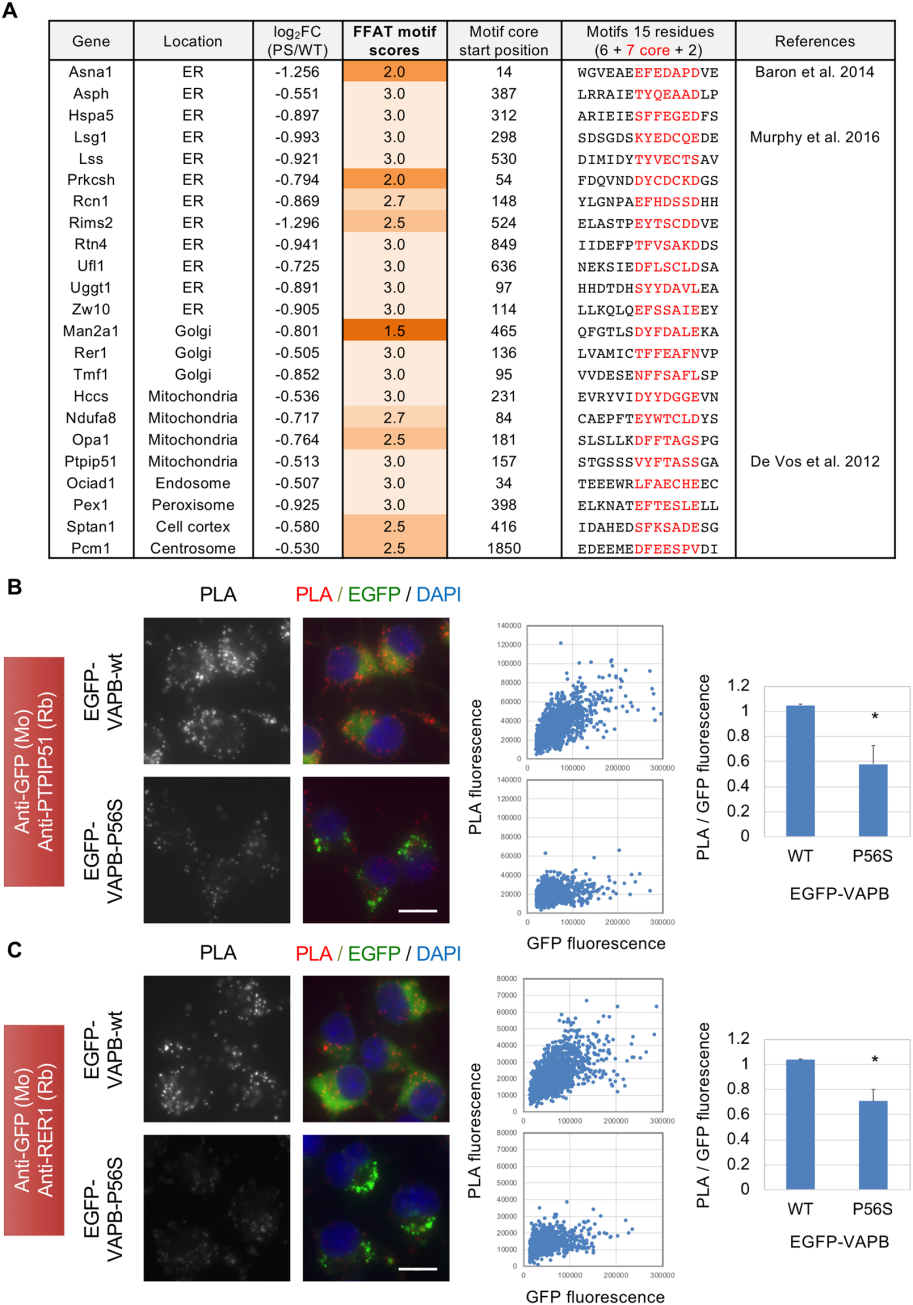
Proximity analysis of EGFP-VAPB to proteins containing FFAT-like motif in N2a cells. (**A**) For 443 proteins reduced in EGFP-VAPB-P56S-IP (Log_2_FCs (EGFP-VAPB-P56S-IP/EGFP-VAPB-wt-IP) were less than −0.5), FFAT-like motifs were identified by the algorithm established by Murphy *et al*.^9^. Twenty-three proteins were shown to contain strong FFAT-like motifs (scores were less than 3) at indicated positions and sequences. Among them, three are reported to interact with VAPB. (**B,C**) N2a cell lines expressing EGFP-VAPB-wt or EGFP-VAPB-P56S were subjected to PLA using antibody for EGFP together with those for PTPIP51 (**B**) or RER1 (**C**). PLA signals and merged views with EGFP and DAPI are shown (left panels, scale bars are 10 μm). Fluorescence intensities for PLA and EGFP in each cell were quantified by cell image analyzer and blotted (middle panels). After normalization with their median values, ratios of PLA to EGFP intensities were calculated. Values are means + SD of three independent experiments (*P < 0.05, Welch’s t test).

The interactions of VPPB with multiple FFAT motif-containing proteins are shown to be critical for ER-organelle interactions^10^. Reduced incorporation of the proteins located in different organelles in VAPB-P56S-IP (Fig. 3E) suggests that these interactions were affected by VAPB mutation. We thus checked existence of FFAT-like motif for the 443 proteins found to be reduced in VAPB-P56S-IP (Fig. 3) by using an algorithm established by Murphy *et al*.^9^. We identified 23 proteins containing strong FFAT-like motifs (scores below 3) (Fig. 7A), three of which were reported to bind to VAPB^9,12,42^. Not only the ER, proteins located to other organelles such as Golgi, mitochondria, endosome and peroxisome were also found (Fig. 7A). Among them, we picked up two proteins, PTPIP51 and RER1, and did PLA using GFP antibody together with their antibodies those were validated by other studies^35,43^ and could detect endogenous proteins by Western blotting although some extra bands were detected (Fig. S9B). PLA signals were observed in the cells expressing EGFP-VAPB-wt (Fig. 7B,C), suggesting its proximal localization with PTPIP51 and RER1. In contrast, the PLA signals were reduced in the EGFP-VAPB-P56S-expressing cells (Fig. 7B,C), suggesting that P56S mutation may reduce proximity of VAPB with the proteins located in other organelles.

### Potential PPI networks containing VAPB and its alteration by the mutation

We finally checked protein-protein interaction (PPI) network among the IP proteins using STRING database^44^. We first picked up top 500 proteins with higher abundancies in both Climp63- and DP1-IPs from N2a cells and then subtracted proteins enriched in DP1-IP or Climp63-IP (Fig. 2) to identify specific PPI network containing ER sheet or tubule proteins, respectively. We found that the PPI network with sheet-enriched proteins contained several PPI clusters composed of the proteins related to Nuclear lumen, Ribonucleoprotein complex, Mitochondrion and ER membrane (Fig. S10A). In contrast, the PPI network with tubule-enriched proteins contained additional cluster composed of ER lumen proteins, whereas the components in nuclear lumen cluster were relatively reduced (Fig. S10B). These are highly compatible with the functional annotations of the proteomics data (Fig. 2E). Interestingly, the sheet-enriched proteins, Climp63 and Canx, were found at center of the ER membrane cluster (Fig. S10A), suggesting they may function as hubs mediating multiple PPIs. On the other hand, the tubule-enriched proteins, DP1 and Rtn3/4, were found at periphery of the ER membrane cluster (Fig. S10B). These positional differences may be involved in the alterations of PPI clusters.

PPI network analysis of top 500 of VAPB-wt-IP proteins again identified five PPI clusters (Fig. S11A). VAPB and VAPA were found at edge of the ER membrane cluster, close to the Mitochondrion cluster, supporting their role in ER-mitochondria tethering. In the PPI network for IP proteins without those decreased in VAPB-P56S-IP (Fig. S11B), proteins in the Mitochondrion cluster were reduced, possibly due to disturbance of the ER-mitochondria tethering by the mutation. In addition, a cluster of ER lumen proteins was unobserved. These are similar to the PPI network with sheet-enriched proteins (Fig. S10A) and may reflecting delocalization of VAPB from ER tubule by the mutation.

## Discussion

In this study, we conducted proteomic characterization of the ER domains containing VAPB wild type (wt) and its P56S mutant. For this purpose, we first identified ER sheet- and tubule-specific proteomes. By using these proteomes as references, we further characterized the VAPB proteomes and found decreased tubular property by ALS-linked VAPB mutation. Biochemical and immunofluorescence analyses suggest delocalization of VAPB from ER tubules, which was confirmed by PLA. Our proteomic and PLA analyses further suggest reduced VAPB interaction with the proteins in other organelles. Taken together, our data supports the altered ER domain properties and impaired ER-organelle tethering by the mutation, which may contrast with the gain of its function reported by several previous studies.

We here optimized the strategy for proteomics of different ER domains. We found that ER domains containing Climp-63 and DP1 were exclusively distributed in N2a cells and differentially isolated by immunoprecipitation in a detergent-free condition. Following proteomics identified ER sheet- and tubule-specific proteomes composed of the proteins with different functional annotations, which were conserved among different cell types. In addition to the known domain-enriched proteins, our tubule proteomics identified several proteins with specific cellular functions. Among them, Lnpk is shown to stabilize three-way junctions of ER tubules^45^, and Fam134C-related protein Fam134B mediates ER membrane curvature and ER-phagy^46,47^. Esty1/2 and Pdzd8 function as tethering proteins with plasma membrane and mitochondria, respectively^48,49^. In contrast, ER sheet proteomes contained ER transmembrane proteins with various cellular functions such as protein folding/degradation and lipid/glucose metabolism (Table S1, S3). We also identified multiple non-ER proteins such as nuclear and Golgi proteins in sheet and tubule proteomes, respectively. Incorporation of nuclear proteins is possibly due to the direct connection of ER sheets with nuclear envelop. Golgi protein incorporation may reflect the close localization of ER tubules with Golgi apparatus around centrosome where direct connection could be induced. This also highlights the different properties of ER sheets and tubules in the cells. Further analyses focusing on the proteome components may unveil unidentified ER domain-specific functions.

By utilizing the identified ER sheet and tubule proteomes as references, we performed proteomic characterization of the ER domains containing VAPB and found it had intermediate domain properties. This was confirmed by IP-Western blotting and immunofluorescence analyses, that is, VAPB was contained in both ER sheets and tubules. Interestingly, proteomics further revealed specific reduction of ER tubule but not sheet proteins in the VAPB-P56S-IP, which was supported by IP-Western blotting and Percoll fractionation. We further observed distinct delocalization of VAPB-P56S from ER tubules and its aggregation around ER sheets, which was finally confirmed by PLA. Notably, distinct accumulation of Climp63 to the mutant VAPB aggregates was not observed (Fig. 5B), suggesting aggregates were not directly contained in the ER sheets, which is consistent with the previous observation in neurons of mutant VAPB transgenic mice^25^. Additional no containing of tubule proteins in the aggregates suggest other structural organizers involved in ER clustering. One candidate would be Canx because it showed relative accumulation to the aggregates (Fig. 5B) and is shown to induce de-novo generation of ER sheets when overexpressed in cultured cells^50^, as observed for Climp63^31^. Proteomics also revealed reduced incorporation of the proteins related to mitochondria, Golgi and peroxisome in the VAPB-P56S-IP, some of which were shown to contain strong FFAT-like motifs. Finally, reduced interactions with some of these were supported by PLA. Our comprehensive analyses thus support loss of tethering function of mutant VAPB, rather than its gain of toxic functions previously reported^12,14,16^. However, it should be necessary to examine whether ER interaction with the organelles are actually affected by the mutation. Furthermore, consequence of the disturbed ER-organelle contacts on cellular functions should be examined in future analysis.

PPI network analysis identified several PPI clusters composed of proteins in different cellular components. VAPB/A, ER sheet and tubule proteins were all contained in the ER membrane cluster but notably located in different regions; VAPB/A at edge close to mitochondrion cluster, and sheet and tubule proteins at the center and periphery, respectively, which may generate the differences in the PPI clusters. Especially, this analysis again highlights the role of VAPB in ER-mitochondria tethering, which may be affected by the mutation. Loss of ER lumen cluster may reflect the delocalization of VAPB from ER tubule, however direct link connecting these two alterations is not apparent in the PPI networks, implying independence of these effects caused by the mutation. It should be noted that proteins without PPIs may be incorporated to some extent because of no use of detergents in the IPs. Thus, the PPI networks should be re-evaluated by further detailed analysis. Our MS analysis also identified potential amino acid modifications such as phosphorylation and ubiquitination in IP proteins (Table S1–6), however clear differences were so far not found among the IP samples possibly because of relative low abundancies of the modified peptides. Deeper sequencing and/or pre-concentration of modified peptides may be necessary for further discussion.

In summary, our established technique identified the ER sheet/tubule-specific proteomes, which enable us to clarify the deranged domain properties in disorganized ER by VAPB mutation. Our data suggest VAPB delocalization and reduced ER-organelle interactions by VAPB mutation, which could be involved in neuronal dysfunction in ALS disease pathogenesis. Notably, reduction of ER-mitochondria contacts is reported for ALS with mutations in other genes, SOD1 and SIGMAR1^51^, suggesting that dysregulation of ER-organelle interaction could be a common pathomechanism in ALS. Other than ALS, formation of ER inclusions is also reported for several neurological diseases including torsion dystonia-1 and Charcot-Marie-Tooth disease^52–54^. In addition, we recently found that neuron-specific knockdown of NF-Y, a ubiquitous transcription factor affected in Huntington’s disease^55^, induces formation of ER inclusions containing insolubilized various membrane proteins^56,57^. Future proteomic studies focusing on theses disorganized ERs may clarify novel aspects on pathogenesis of several neurological diseases.

## Methods

### Antibodies

Antibodies for Climp63/CKAP4 (16686-1-AP), DP1/REEP5 (14643-1-AP), VAPB (14477-1-AP), VAPA (15275-1-AP), PTPIP51 (20641-1-AP) were obtained from Proteintech; antibodies for RFP (clone 3G5; M165-3, clone 8D6; M155-3), EGFP (598, D153) were obtained from MBL; an antibody for Rtn4A/Nogo A (sc-25660) was obtained from Santa Cruz; antibodies for Canx (SPA-860), KDEL (PM059) were obtained from Enzo; an antibody for GM130 (610822) was obtained from BD, an antibody for RER1 (HPA051400) was obtained from Atlas Antibodies, an antibody for EGFP (1814460) was obtained from Roche. Western blot data for principal antibodies are shown in Fig. S9.

### Plasmids

The cDNA for human VAPB (wild type (wt)) was cloned by standard PCR methods from a cDNA mixture prepared from Human Brain total RNA (Clontech) using ThermoScript RT-PCR Systems (Thermo Fisher Scientific). Obtained cDNA was subcloned into pcDNA5-FRT-TO vector (Thermo Fisher Scientific) containing EGFP sequence to generate stable cell lines. VAPB-P56S mutant was generated by PCR based mutagenesis using two primers, AGGTACTGTGTGAGGTCCAACAGCGGAATCA and TGATTCCGCTGTTGGACCTCACACAGTACCT. The mouse cDNAs for Climp63 and DP1 were cloned by standard PCR methods from a mouse brain cDNA mixture, and then subcloned into pcDNA5-FRT-TO vector containing EGFP or mRFP sequence.

### Cell lines and culture

Cultured cell lines were maintained in DMEM supplemented with 10% FBS, penicillin–streptomycin in an atmosphere containing 5% CO2. Flp-in Neuro2a (N2a/FRT/TR) cell line was derived from N2a mouse neuroblastome cells^58,59^. Flp-in HEK293 cell line was purchased from Thermo Fisher Scientific. The N2a and HEK293 inducible cell lines expressing various ER membrane proteins under doxycycline were generated using Flp-in system (Thermo Fisher Scientific); the flip-in cells were transfected with above pcDNA5-FRT-TO vectors together with pOG44, and stable cell lines were obtained by culturing the cells in growth medium containing 15 μg/ml Blasticidin and 100 μg/ml Hygromycin. Transfection was performed using lipofectamine 2000 (Invitrogen) according to the manufacture’s protocol.

### Immunofluorescence microscopy

Cells were fixed with 3.7% formalin/phosphate-buffered saline (PBS) and blocked with 0.1% Triton-X 100/5% goat serum in Tris-buffered saline-0.05% Tween20 (TBST). The fixed cells were then incubated with primary antibodies in 0.1% bovine serum albumin (BSA)/TBST followed by with secondary antibodies conjugated with Alexa fluorescent dyes (Thermo Fisher Scientific) in the same buffer. After mounting with a VECTASHIELD mounting medium containing DAPI (VECTOR), fluorescence images were obtained on a FV1000 confocal system (Olympus). Fluorescence intensities were measured using RBG profile plot in Image J software^60^.

### Cell fractionation and immunoprecipitation

For fractionation, cells were suspended in ~300 ul of HB buffer (0.25 M Sucrose, 20 mM HEPES pH7.2, 1 mM EDTA and 0.5X cOmplete (Roche)), and passed through 29 G needle for 30 times. After measuring protein concentration, the homogenates with equal protein concentration were briefly sonicated for 30 sec by a Bioruptor sonicator (BMBio). For immunoprecipitation the homogenates were centrifuged at 1000 g or 6000 g for 10 min at 4 °C. The supernatants (inputs; 100 μl) were diluted with 400 μl of PBS with 0.5X cOmplete (Roche), and were incubated with antibodies for mRFP (3G5 and 8D6) or EGFP (598 and D153) for 2 hr at 4 °C, followed by incubation with Protein A/G magnetic beads (Pierce) for 2 hr. After adding 1 ml of 0.01% BSA/PBS, the beads were washed with 0.5 ml of 0.01% BSA/PBS and then with 1 ml of PBS twice. Immunoprecipitates were eluted with urea buffer (8 M urea, 50 mM Tris-HCl, pH 7.5, 10 mM DTT) or Hepes-SDS buffer (100 mM Hepes, pH 8.0, 1% SDS, 10 mM DTT) at 95 °C for 5 min. For protein detection, a part of the samples was mixed with SDS sample buffer, and subjected to SDS-PAGE followed by Western blotting as described previously^61^. Chemiluminescent signals were obtained and quantified using ImageQuant LAS-4000 (GE).

### Percoll density gradient fractionation

Total cell homogenates prepared above (100 μg of protein in 20 μl of HB buffer) were diluted in 1 ml 30% Percoll/HB buffer, and ultra-centrifuged at 60000 g (36700 rpm; Beckman MLA-150 rotor) for 30 min at 4 °C. Fractions (50 or 100 μl) were collected from the top, mixed with equal volume of SDS sample buffer and heated at 100 C for 5 min. After centrifuging at 20,000 g for 30 min, supernatants were subjected to Western blotting.

### Protein digestion and LC-MS analysis

Protein digestion was performed with Filter-aided Sample Preparation (FASP) method^62^. Immunoprecipitates and inputs in urea or Hepes-SDS buffer (10 μl) were diluted with 90 μl of 8 M urea/100 mM Tris-HCl (pH 8.0). After adding 10 μl of 333 mM iodoacetamide (final 30 mM), the mixture was incubate at room temperature for 30 min in the dark, and then filtered with Ultrafiltration device (mwco 30 kDa; Aproscience) by centrifuging at 14,000 g for ~10 min. The trapped proteins on the filter were washed with 300 μl of 100 mM NH_4_HCO_3_ for four times, then digested with 40 μl of 0.2 μg/μl Modified Trypsin (Promega V511A) in 100 mM NH_4_HCO_3_ at 37 °C overnight. The digested proteins were collected by centrifuging the filter unit after adding total 80 μL of 100 mM NH_4_HCO_3_ and then 50 μL of 0.5 M NaCl. The filtrates were acidified by trifluoroacetic acid, desalted with C18 StageTip and SCX Tip, and then applied to the liquid chromatography/mass spectrometry (LC-MS) system. Briefly, the peptides were separated by a EASY-nLC 1000 (Thermo Fisher Scientific), ionized with nano-ESI, and analyzed using a QExactive hybrid quadrupole-orbitrap mass spectrometer (Thermo Fisher Scientific) to obtain MS/MS spectrum data of the peptides. Based on this peptide information, proteins were identified using Proteome Discoverer version 2.2 (PD2.2, Thermo Scientific) with the MASCOT search engine software (Matrix Science). For data analysis, PD2.2 was used to quantify abundancies of the proteins (label free quantification)^34^. After normalization with median values of protein abundancies and/or the amount of precipitated EGFP/mRFP-tagged proteins, the proteins with low abundancies and those found in control IPs (non-specifically precipitated proteins) were excluded. Protein abundancies were then compared between the two IP sets and log2 values of the fold changes (FCs) were calculated. Statistical analysis was performed by one-way ANOVA followed by Tukey post-test using Prism software. Identified proteins were listed in Supplementary Tables. Potential modifications including acetylation, phosphorylation and ubiquitination (di-glycine modified lysine) searched by PD2.2 were also listed in these tables. The identified proteins were processed for functional annotation using the DAVID bioinformatics database^63^, and P < 0.05 was considered significant.

### PPI network analysis

To identify specific PPI network containing ER sheet or tubule proteins, we first picked up top 500 proteins with higher abundancies in both Climp63- and DP1-IPs from N2a cells, and then proteins enriched in DP1-IP or Climp63-IP were subtracted before analysis, respectively. For PPI networks in VAPB-IP proteins, we first analyzed top 500 proteins with higher abundancies in VAPB-wt-IP from N2a cells, and then analyzed those without the proteins decreased in VAPB-P56S-IP to clarify the effect of VAPB mutation on the PPI network. These proteins were analyzed by STRING database^44^ through following settings; organism, Homo sapiens; active interaction sources, Experiments, Databases and Co-expression; minimum required interaction score, 0.4; interactions, query proteins only. Proteins found in enriched GO cellular components including nuclear lumen (GO:0031981), ribonucleoprotein complex (GO:1990904), mitochondrion (GO:0005739), ER membrane (GO:0005789) and ER lumen (GO:0005788) were color-labeled.

### Immunofluorescence cell image analysis

Immunofluorescence cell data of the proteins identified by IP-proteomics were obtained from the Cell Atlas in the Human Protein Atlas database^35,36^. Fluorescence signals (green) from central to peripheral regions (1~4) were quantified using ImageJ software^60^, and relative fluorescence intensities (mean intensities + SD for three cells) were obtained.

### Identification of FFAT-like motif

Mouse whole peptide sequences (Mus_musculus.GRCm38.pep.all.fa.gz) were obtained from Ensembl database, and the longest peptide sequences for 443 proteins reduced in EGFP-VAPB-P56S-IP (Log2FCs (EGFP-VAPB-P56S-IP/EGFP-VAPB-wt-IP) < −0.5) were obtained using SeqKit software^64^. FFAT-like motifs were identified by the algorithm established by Murphy *et al*.^9^, and the sequences with less than 3 scores were considered as strong FFAT-like motifs.

### Proximity ligation assay (PLA)

PLA^40,41^ was performed using Duolink *In Situ*-Fluorescence (Sigma-Aldrich) according to the manufacture’s protocol. Briefly, cells expressing EGFP-VAPB-wt or EGFP-VAPB-P56S were cultured in 8-well chamber slide. Then, the cells were fixed with 3.7% formalin/PBS at room temperature for 20 min and blocked with 0.1% Triton-X 100/5% goat serum in TBST for 10 min. The fixed cells were then co-incubated with mixed antibodies derived from mouse or rabbit in 0.1% BSA/TBST at 4 °C overnight, followed by incubation with PLA probes (anti-Mouse PLUS and anti-Rabbit MINUS) at 37 °C for 1 hr. After ligation and amplification using Detection Reagents ORANGE, the cells were washed with Wash Buffer B, and then mounted with Duolink *In Situ* Mounting Medium with DAPI. Fluorescence images for PLA, EGFP and DAPI were randomly obtained on a BZ-X710 microscope (Keyence, Osaka, Japan). Fluorescence intensities for PLA and EGFP in each cell were quantified by using Thermo Scientific CellInsight NXT image analyzer. After normalization with their median values, we chose the cells with over 5000 of EGFP fluorescence intensities and calculated ratios of PLA to EGFP intensities. Because the variances were not equal by F-test, the data were analyzed by Welch’s t test (two-tailed).

## Supplementary information

Supplementary information.

Supplementary Tables
