# Supplementary material for: Proteomics-Based Approach Identifies Altered ER Domain Properties by ALS-Linked VAPB Mutation
Source: Sci Rep. 2020 May 6;10:7610. doi: 10.1038/s41598-020-64517-z (PMC7203144; doi:10.1038/s41598-020-64517-z)
Supplement: Supplementary file 1 — Supplementary information. [file 41598_2020_64517_MOESM1_ESM.pdf]

Proteomics-Based Approach Identifies Altered ER Domain Properties by ALS-Linked VAPB Mutation.

Tomoyuki Yamanaka, Risa Nishiyama, Tomomi Shimogori and Nobuyuki Nukina

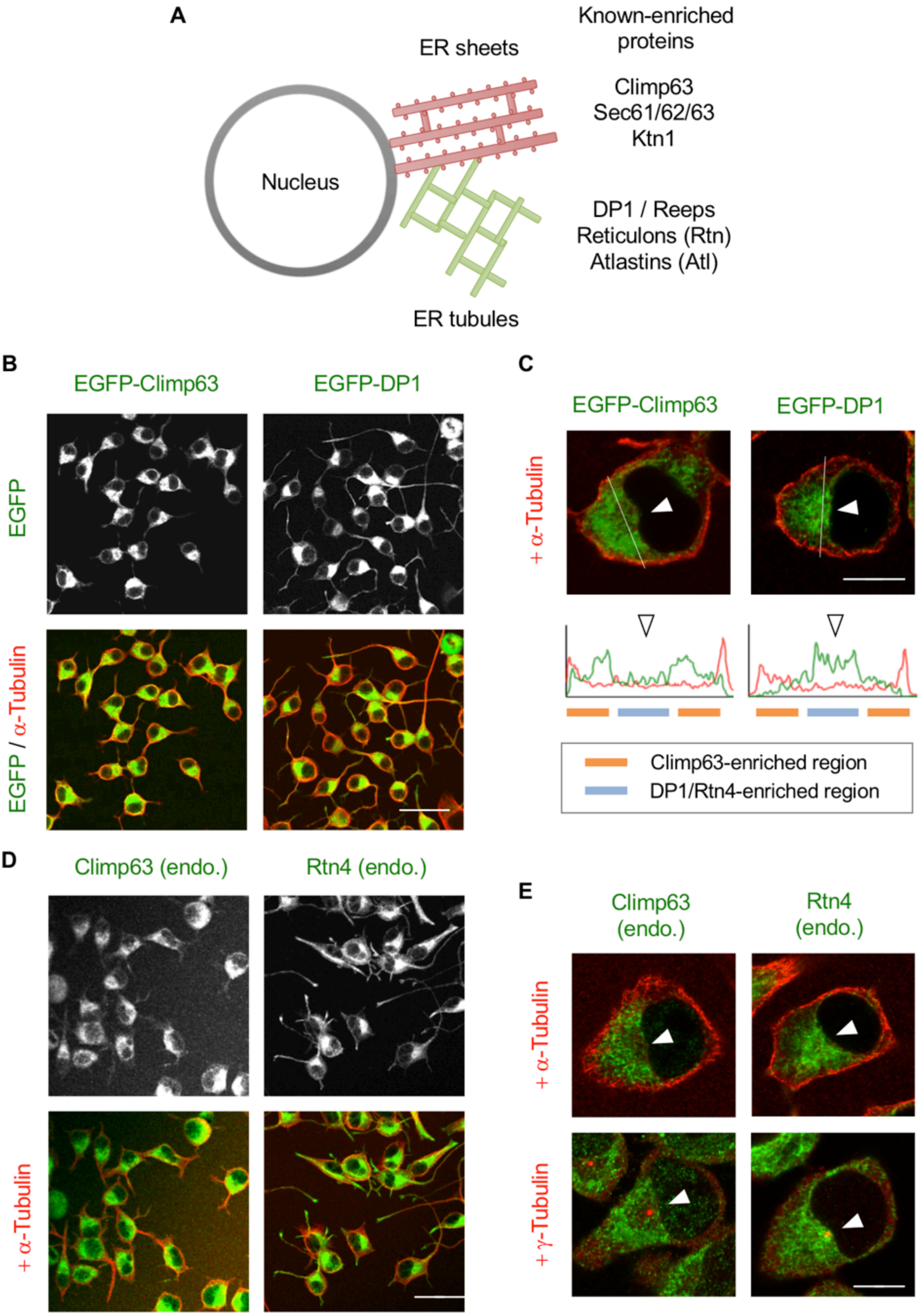
A
Known-enriched proteins
ER sheets
Nucleus
Climp63
Sec61/62/63
Ktn1
DP1 / Reeps
Reticulons (Rtn)
Atlastins (Atl)
ER tubules
B
EGFP-Climp63
EGFP-DP1
EGFP
EGFP / α-Tubulin
C
EGFP-Climp63
EGFP-DP1
+ α-Tubulin
Climp63-enriched region
DP1/Rtn4-enriched region
D
Climp63 (endo.)
Rtn4 (endo.)
+ α-Tubulin
E
Climp63 (endo.)
Rtn4 (endo.)
+ α-Tubulin
+ γ-Tubulin

Supplementary Figure S1. Generation of N2a cells expressing ER sheet / tubule proteins.

A. Scheme of the ER sheets and tubules and list of the proteins known to be differentially accumulated to these ER domains. B. N2a cell line expressing EGFP-Climp63 or EGFP-DP1 (green) was stained with α-tubulin (red). Note that EGFP-Climp63 was preferentially localized in cell bodies whereas EGFP-DP1 was localized in perinuclear as well as neurites positive for α-tubulin. C. Higher magnification of the localizations of EGFP-Climp63 and EGFP-DP1. They showed different distributions in cell bodies; spatial accumulation of EGFP-DP1 at perinucleus (indicated by arrowheads) whereas cell body localization of EGFP-Climp63 outside of it. Fluorescence intensities of the regions indicated by white lines are plotted on the bottom of the panels, and the regions enriched for Climp63 and DP1 are indicated by orange and blue lines, respectively. D. Parental N2a cells were stained with antibodies against Climp63 and Rtn4 (green) together with α-tubulin (red). Endogenous Climp63 and Rtn4 showed similar distributions with EGFP-Climp63 and EGFP-DP1, respectively. E. Higher magnification of the cell body stains for endogenous Climp63 and Rtn4 (green) co-stained with α- and γ-tubulins (red). Endogenous Rtn4 was accumulated to perinucleus positive for γ-tubulin whereas Climp63 was located in cell bodies outside of it. Scale bars are 40 μm (B, D) and 10 μm (C, E).

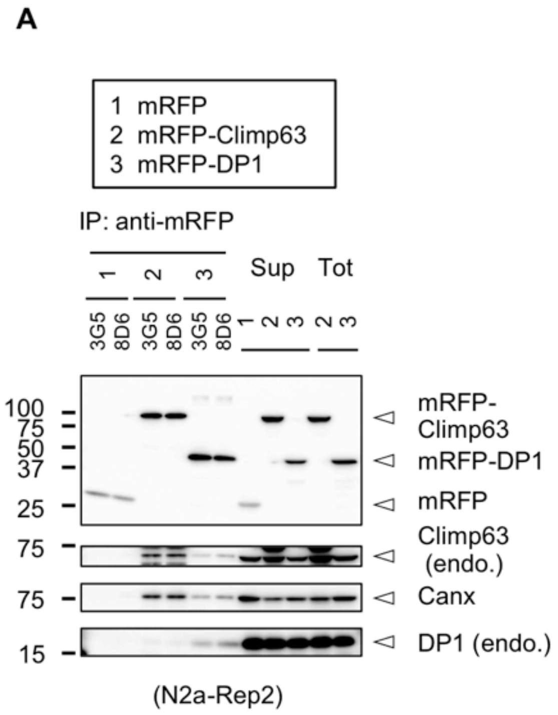

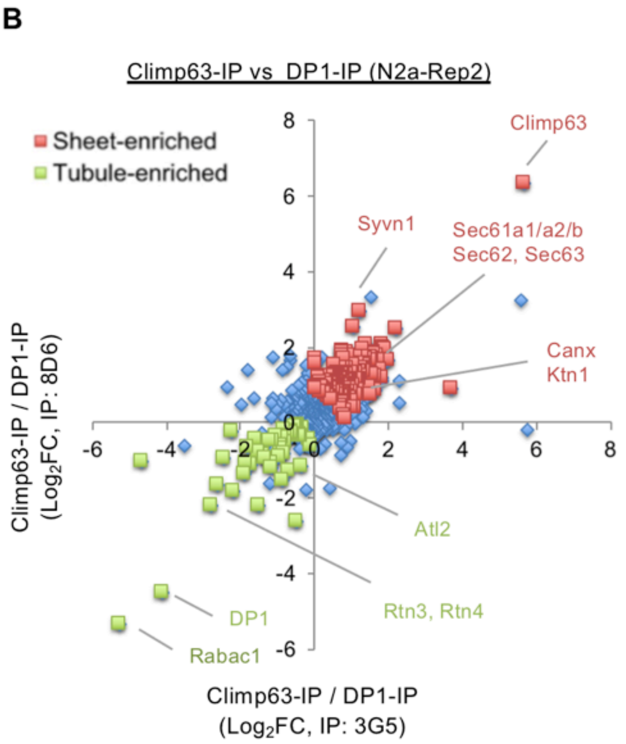

Supplementary Figure S2. Proteomic analysis of the immunoprecipitates for mRFP-Climp63 and mRFP-DP1 from N2a cells (replicate 2).

A. Western blotting of the immunoprecipitates by anti-mRFP antibodies (3G8 and 8D6) from N2a cell lines expressing mRFP, mRFP-Climp63 and mRFP -DP1. Precipitated proteins were analyzed by antibodies for indicated proteins. B. Immunoprecipitates were digested with trypsin and analyzed by LC-MS. Proteome Discoverer software 2.2 was used to identify the proteins and quantify their abundancies (label free quantification). After normalization with the amount of precipitated mRFP-tagged proteins, log2 values of the fold changes (FCs; mRFP-Climp63-IP / mRFP-DP1-IP) were calculated and plotted for two IP sets (3G5 and 8D6). Proteins enriched by mRFP-Climp63-IP (red) or mRFP-DP1-IP (green) in two biological replicates (Figure 2D) were colorized and known sheets and tubule proteins are indicated.

A

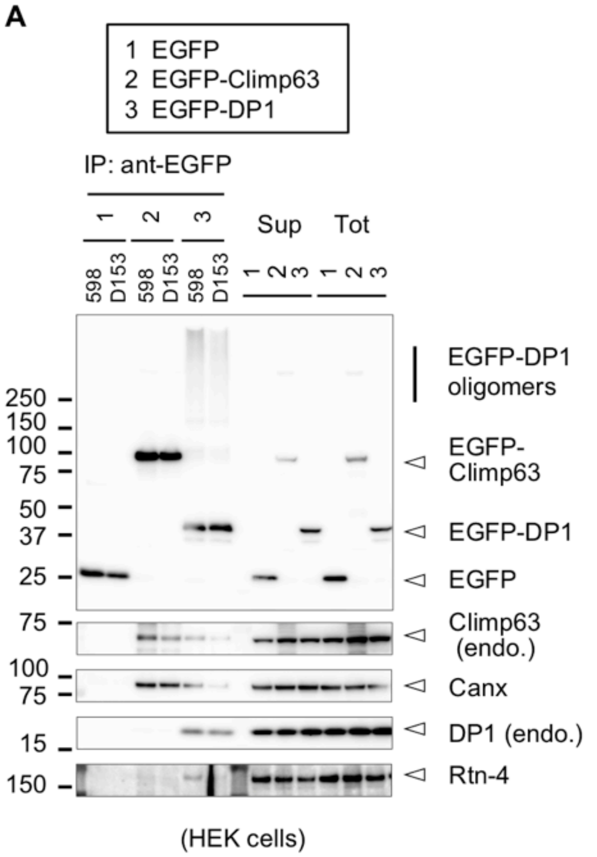

B

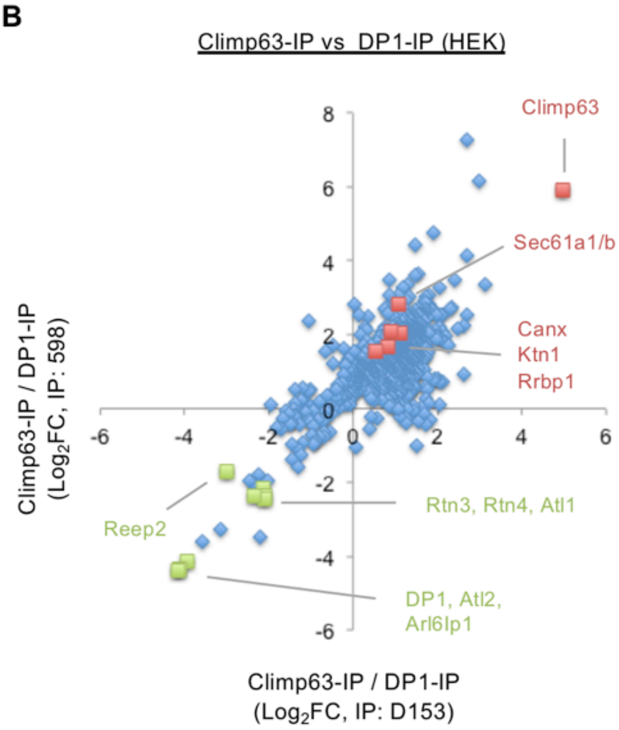

C

HEK: Climp63-IP > DP1-IP (536 proteins)			
Term	Count	PValue	Fold Enrichment
Ribonucleoprotein	88	5.21E-69	11.95
Nucleus	308	5.07E-63	2.36
RNA-binding	96	3.16E-45	5.80
Ribosomal protein	56	7.46E-44	12.17
Chromosome	53	7.02E-23	5.33
ER	43	2.26E-03	1.62
ER membrane	40	2.08E-03	1.66

HEK: Climp63-IP < DP1-IP (52 proteins)			
Term	Count	PValue	Fold Enrichment
ER	36	5.60E-38	17.06
ER membrane	22	1.91E-18	12.69
ER lumen	11	4.84E-14	43.33
Smooth ER	5	6.51E-07	70.63
Transmembrane	29	3.83E-05	1.97
Protein folding	5	2.61E-04	15.70
ERGIC	4	5.75E-04	24.10

D

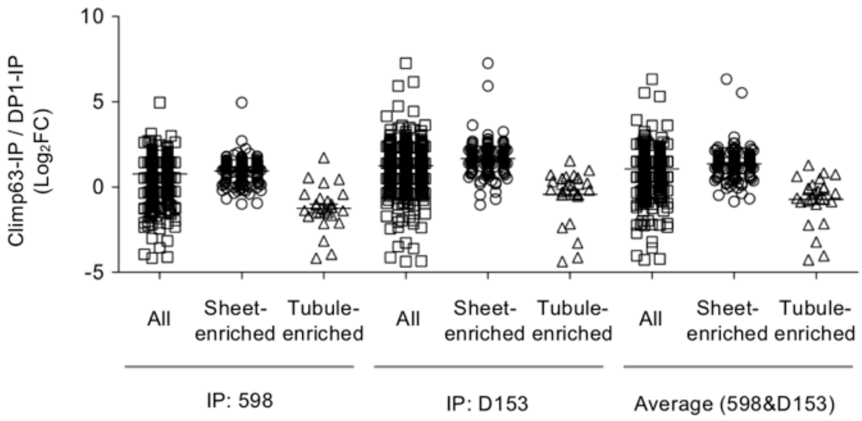

Supplementary Figure S3. Proteomic analysis of the immunoprecipitates for EGFP-Climp63 and EGFP-DP1 from HEK293 cells.

A. Western blotting of the immunoprecipitates by anti-EGFP antibodies (598 and D153) from HEK293 cell lines expressing EGFP, EGFP-Climp63 and EGFP-DP1. Precipitated proteins were analyzed by antibodies for indicated proteins. Note the preferential precipitation of endogenous Climp63 and Canx in the EGFP-Climp63-IP whereas endogenous DP1 and Rtn4 in the EGFP-DP1-IP. B. Immunoprecipitates were digested with trypsin and analyzed by LC-MS. Proteome Discoverer software 2.2 was used to identify the proteins and quantify their abundancies (label free quantification). After normalization with the amount of precipitated EGFP-tagged proteins, log2 values of the fold changes (FCs; EGFP-Climp63-IP / EGFP-DP1-IP) were calculated and plotted for two IP sets (598 and D153). Known sheets (red) and tubule (green) proteins as well as Canx (red) are indicated. C. Identified proteins from HKE293 cells were processed for functional annotation analysis. Proteins related to nucleus and RNA were relatively abundant in the EGFP-Climp63-IP, whereas proteins related to ER lumen and ER network were abundant in the EGFP-DP1-IP. D. Among the identified proteins by two IP sets from HKE293 cells (All), their FCs of the proteins enriched by mRFP-Climp63-IP (sheet) or mRFP-DP1-IP (tubule) in N2a cells (Figure 2D) were picked up and plotted. Averaged values were plotted on the right.

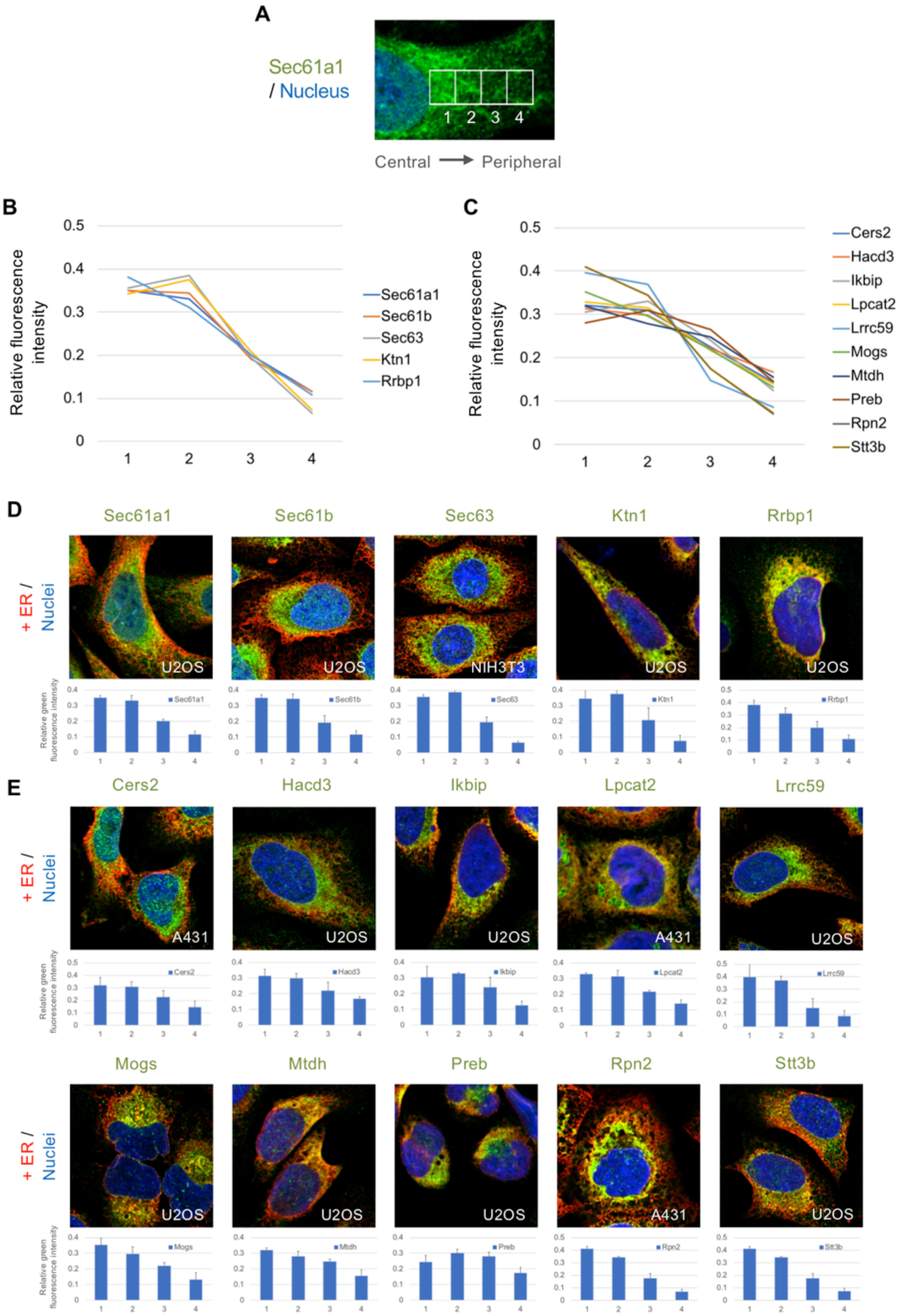
A
Sec61a1
/ Nucleus
1 2 3 4
Central
Peripheral
B
Relative fluorescence intensity
0.5
0.4
0.3
0.2
0.1
0
1 2 3 4
Sec61a1
Sec61b
Sec63
Ktn1
Rrbp1
C
Relative fluorescence intensity
Cers2
Hacd3
Ikbip
Lpcat2
Lrrc59
Mogs
Mtdh
Preb
Rpn2
Stt3b
D
+ ER / Nuclei
Sec61a1
Sec61b
Sec63
Ktn1
Rrbp1
U2OS
NIH3T3
Relative green fluorescence intensity
E
Cers2
Hacd3
Ikbip
Lpcat2
Lrrc59
A431
Mogs
Mtdh
Preb
Rpn2
Stt3b

Supplementary Figure S4. Subcellular distributions of the identified proteins enriched in Climp63-IP.

Immunofluorescence data of the proteins identified by IP-proteomics were obtained from Human Protein Atlas database, and their fluorescence signals (green) from central to peripheral (regions 1~4) were quantified. A. An example image for an ER sheet protein Sec61a1. Quantified regions are indicated. B-C. Relative fluorescence intensities for known ER sheet proteins enriched in Climp63-IP (B) and identified ER membrane proteins enriched in Climp63-IP (C). Values are means of relative intensities for three cells. D-E. Representative cell images and relative fluorescence intensities (means + SD) were shown for known ER sheet proteins enriched in Climp63-IP (D), and identified ER membrane proteins enriched in Climp63-IP (E). Stained cell lines are indicated at bottom right of the panels.

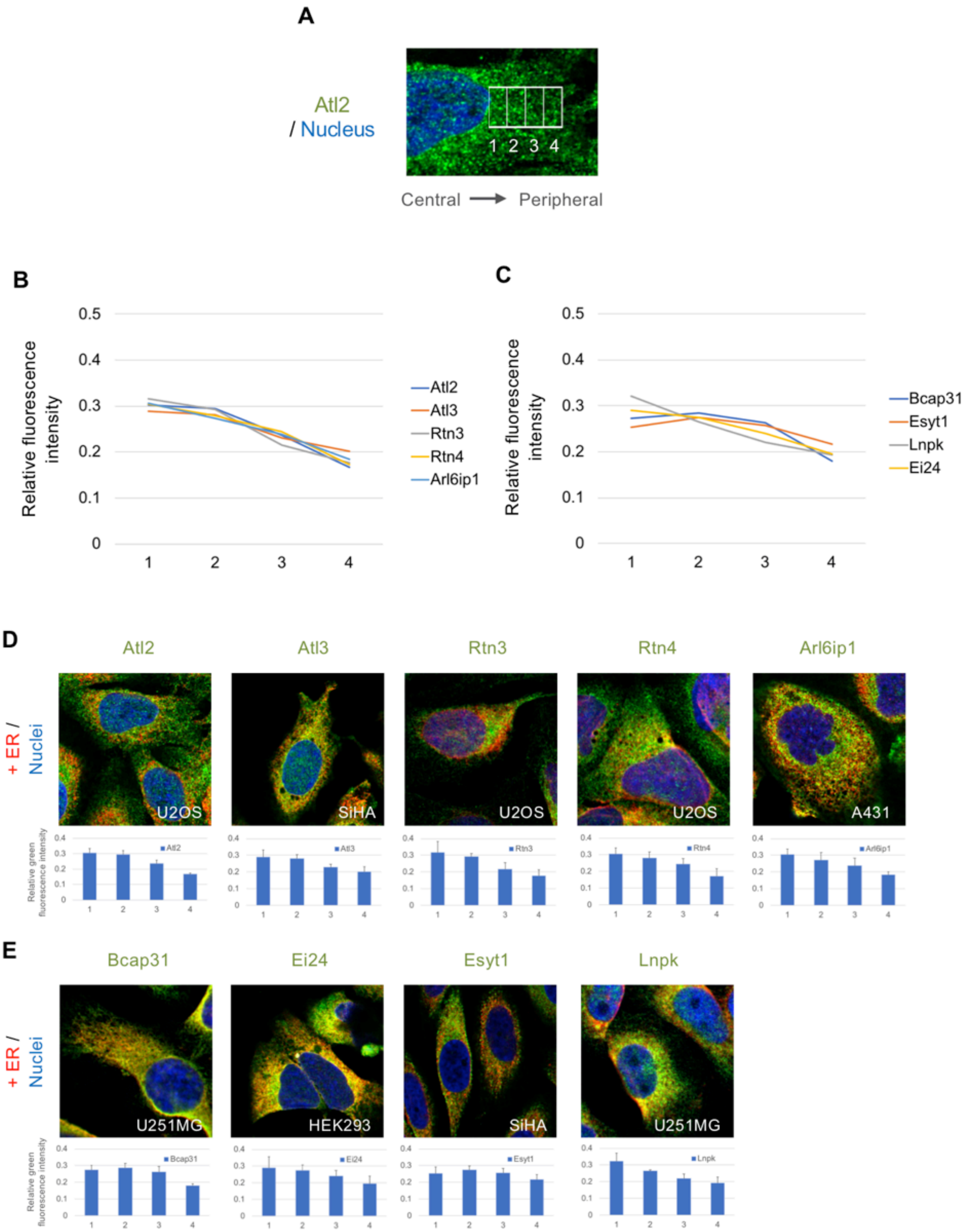

A
Atl2
/ Nucleus
1 2 3 4
Central
Peripheral
B
Relative fluorescence intensity
Atl2
Atl3
Rtn3
Rtn4
Arl6ip1
C
Relative fluorescence intensity
Bcap31
Esyt1
Lnpk
Ei24
D
Atl2
Atl3
Rtn3
Rtn4
Arl6ip1
+ ER / Nuclei
U2OS
SiHA
U2OS
U2OS
A431
Relative green fluorescence intensity
E
Bcap31
Ei24
Esyt1
Lnpk
+ ER / Nuclei
U251MG
HEK293
SiHA
U251MG
Relative green fluorescence intensity

Supplementary Figure S5. Subcellular locations of the identified proteins enriched in DP1-IP. Immunofluorescence data of the proteins identified by IP-proteomics were obtained from Human Protein Atlas database, and their fluorescence signals (green) from central to peripheral (regions 1~4) were quantified. A. An example image for an ER tubule protein Atl2. Quantified regions are indicated. B-C. Relative fluorescence intensities for known ER tubule proteins enriched in DP1-IP (B) and identified ER membrane proteins enriched in DP1-IP (C). Values are means of relative intensities for three cells. D-E. Representative cell images and relative fluorescence intensities (means + SD) were shown for known ER tubule proteins enriched in DP1-IP (D), and identified ER membrane proteins enriched in DP1-IP (E). Stained cell lines are indicated at bottom right of the panels.

A

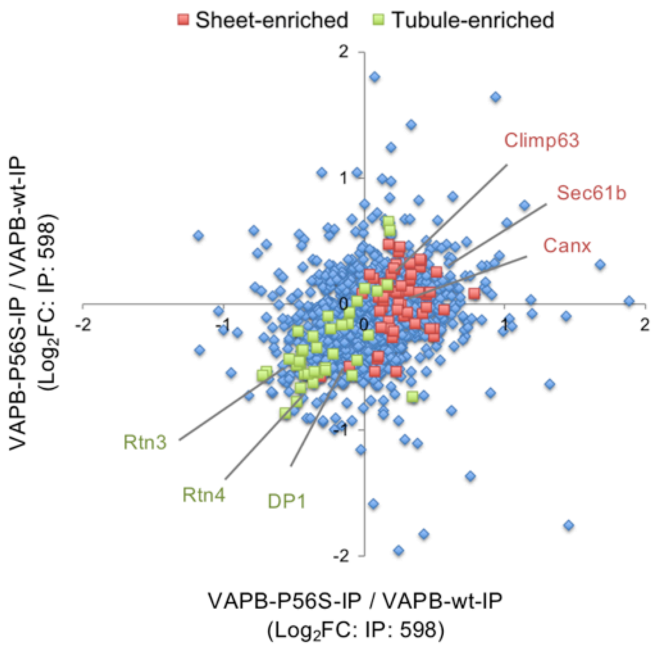

B

VAPB-wt-IP > VAPB-P56S-IP (269 proteins)			
Term	Count	PValue	Fold Enrichment
ER	69	2.03.E-34	6.13
ER membrane	34	1.76.E-10	3.71
ER lumen	20	6.57.E-17	14.89
Smooth ER	10	6.71.E-11	26.69
Mitochondrion	41	1.58.E-11	3.43
RNA-binding	27	5.03.E-09	3.98
ER-Golgi transport	8	6.12.E-05	8.05
Golgi apparatus	19	2.27.E-03	2.24
Peroxisome	7	1.34.E-03	5.80

Supplementary Figure S6. Proteomic analysis of the immunoprecipitates for EGFP-VAPB-wt and its P56S mutant from N2a cells (replicate 2).

N2a cell lines expressing EGFP-VAPB-wt and EGFP-VAPB-P56S were homogenized in detergent-free sucrose buffer and subjected to immunoprecipitation using anti-EGFP antibody (598) (two IP sets for each). Immunoprecipitates were digested with trypsin and analyzed by LC-MS. Proteome Discoverer software 2.2 was used to identify the proteins, quantify their abundancies (label free quantification). A. Protein abundancies were compared between the EGFP-VAPB-wt-IP and EGFP-VAPB-P56S-IP, and Log_2FCs (EGFP-VAPB-P56S-IP / EGFP-VAPB-wt-IP) were plotted. Labeling of the proteins enriched in ER sheets and tubules (Table S1, S2) revealed preferential reduction of the tubule proteins in the EGFP-VAPB-P56S-IP. B. Identified 269 proteins reduced in EGFP-VAPB-P56S-IP were processed for functional annotation analysis. Proteins related to nucleus and ER lumen, mitochondria, Golgi apparatus and peroxisome were relatively enriched.

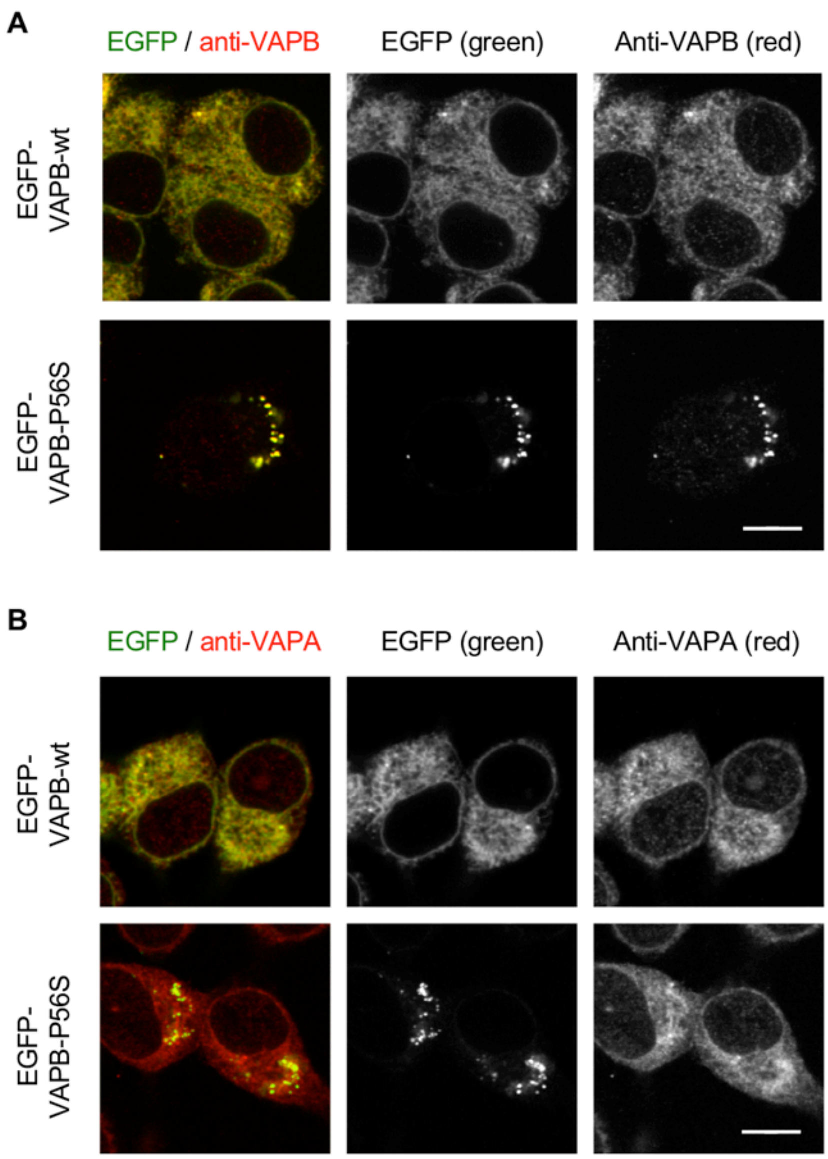

Supplementary Figure S7. Anti-VAPB and VAPA staining of N2a cells expressing EGFP-VAPB-wt and its P56S mutant.

N2a cell line expressing EGFP-VAPB-wt (upper panels) or EGFP-VAPB-P56S (lower panels) (green) was stained with anti-VAPB (A) or anti-VAPA (B) antibody (red).

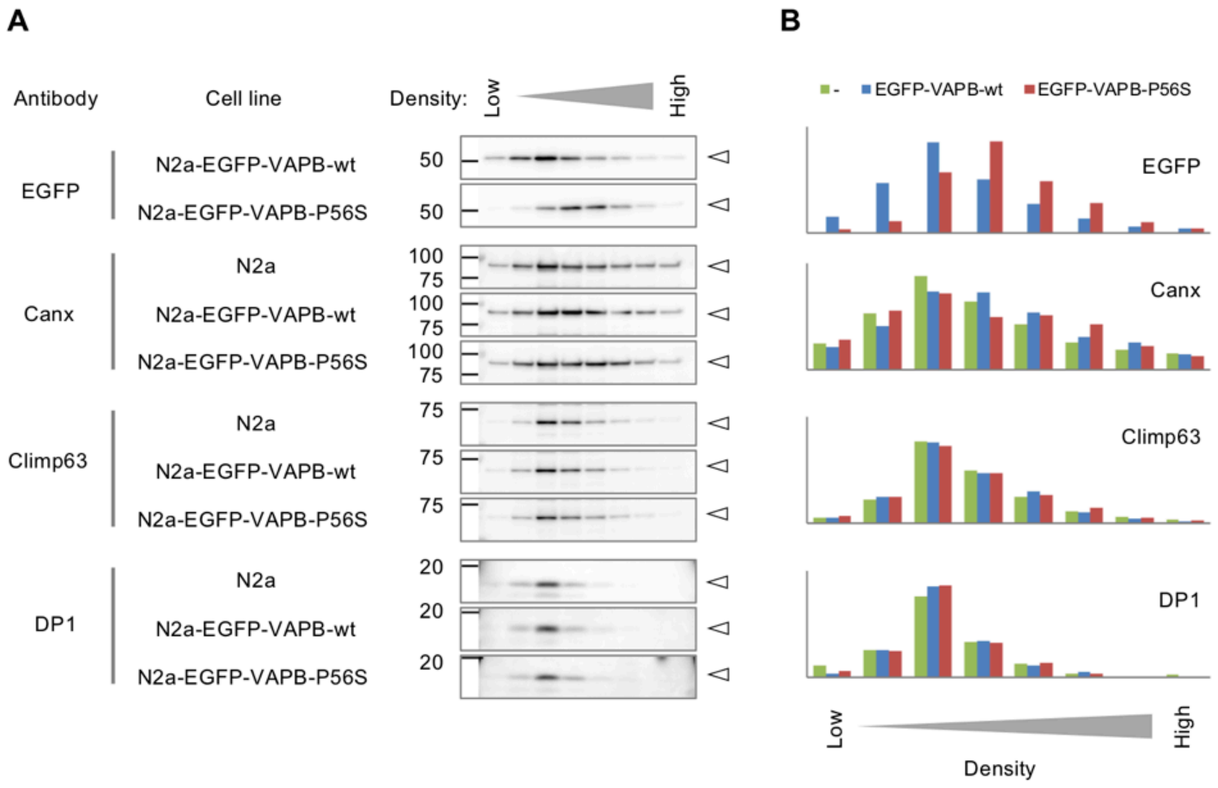

Supplementary Figure S8. Percoll fractionation of N2a cells and those expressing EGFP-VAPB-wt or its P56S mutant (replicate 2).

A. N2a cells or those expressing EGFP-VAPB-wt or EGFP-VAPB-P56S homogenized in detergent-free sucrose buffer were subjected to ultracentrifugation in 30% Percoll / sucrose buffer. Ten fractions were collected from the top, and fractions 2-9 were analyzed by Western blotting for indicated proteins. B. Quantification of the fractionated proteins in A. Values are one or means of two data.

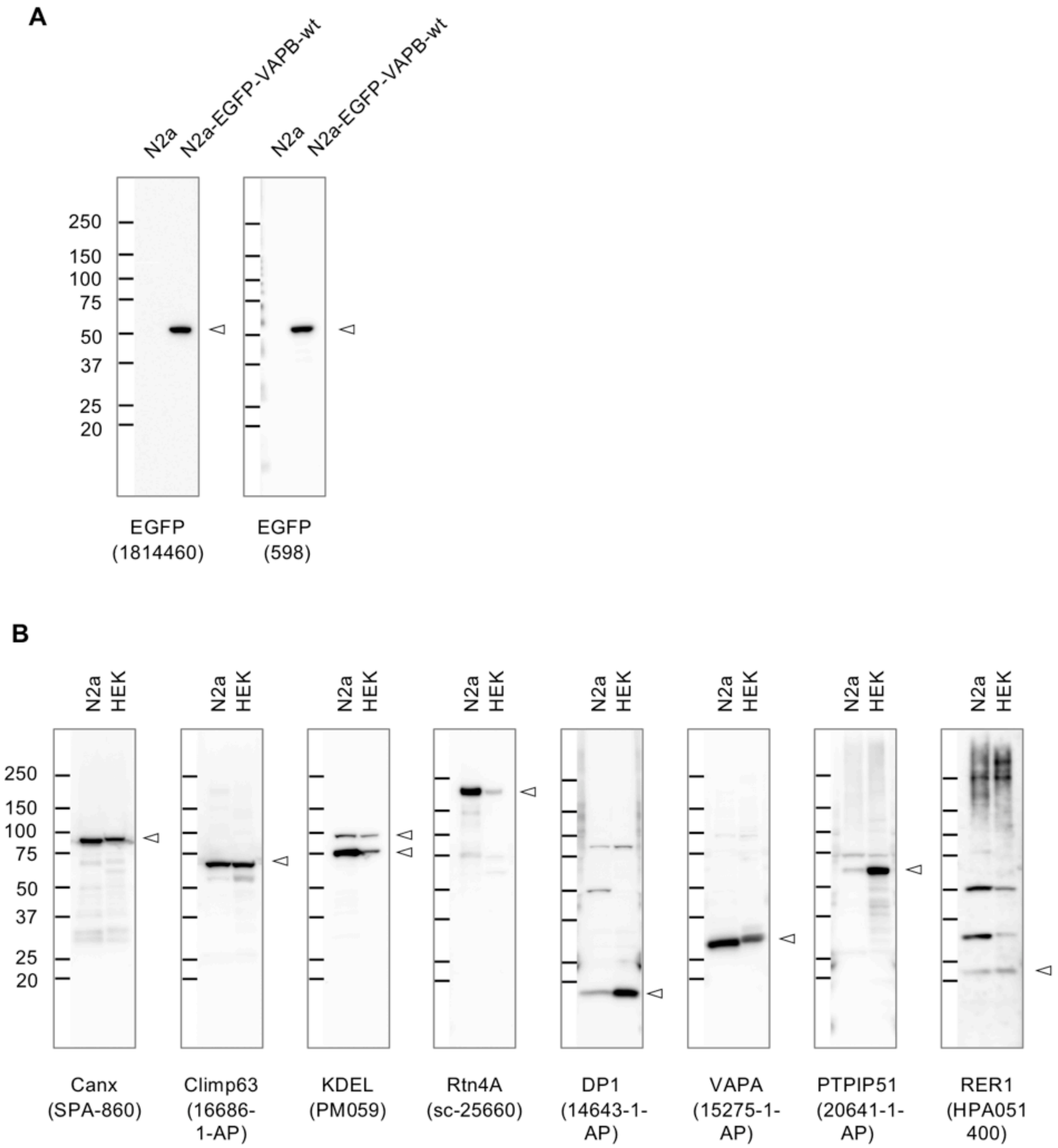

Supplementary Figure S9. Western blotting of total cell homogenates.

A. Total homogenates of N2a cells or those expressing EGFP-VAPB-wt were subjected to Western blotting using anti-EGFP mouse (1814460) and rabbit (598) antibodies. Arrowheads indicate the bands for EGFP-VAPB-wt protein. B. Total homogenates of N2a and HEK cells were subjected to Western blotting using antibodies for indicated proteins. Arrowheads indicate the bands for corresponding endogenous proteins.

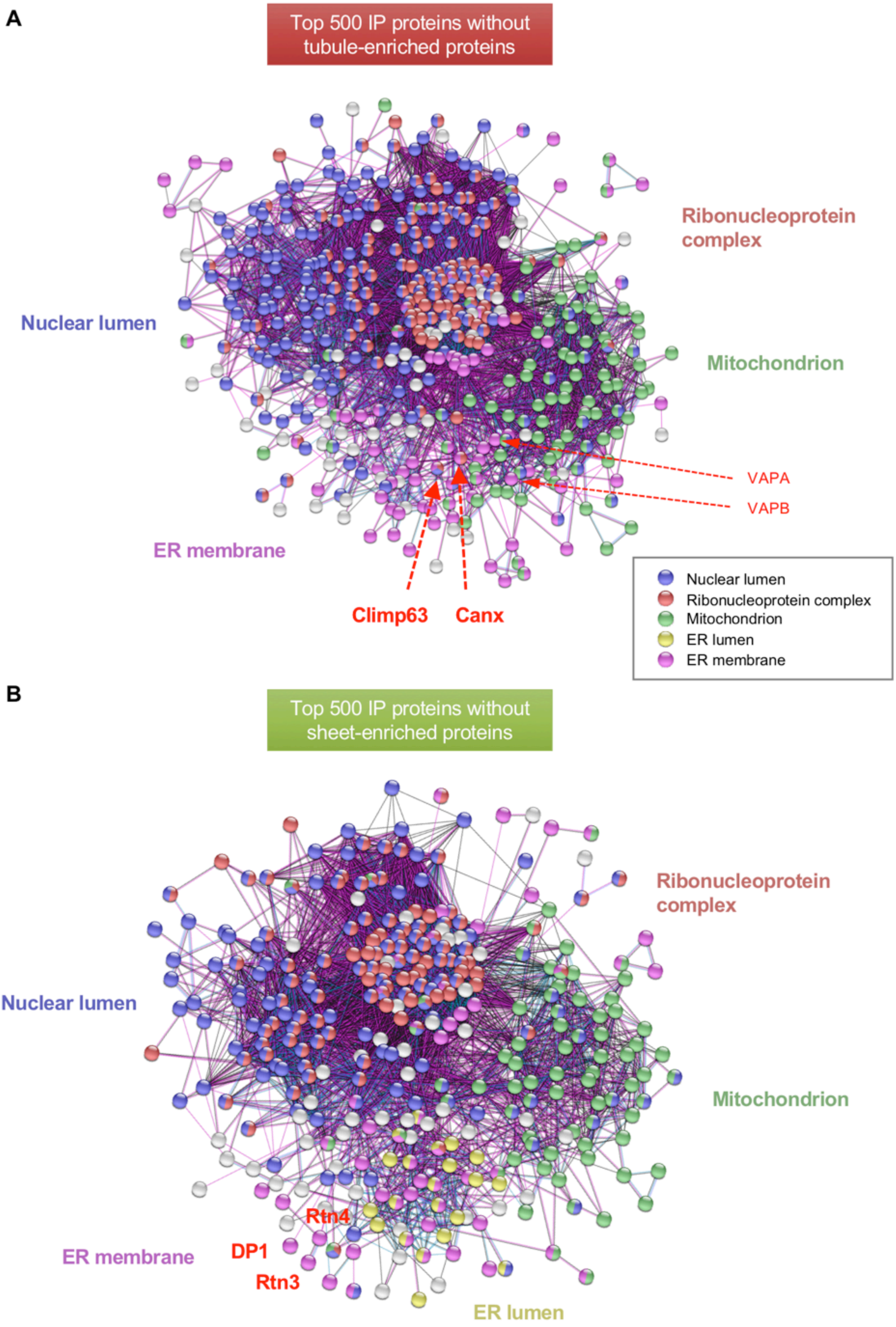
A
Top 500 IP proteins without tubule-enriched proteins
Ribonucleoprotein complex
Nuclear lumen
Mitochondrion
VAPA
VAPB
ER membrane
Climp63
Canx
Nuclear lumen
Ribonucleoprotein complex
Mitochondrion
ER lumen
ER membrane
B
Top 500 IP proteins without sheet-enriched proteins
Ribonucleoprotein complex
Nuclear lumen
Mitochondrion
Rtn4
DP1
Rtn3
ER membrane
ER lumen

Supplementary Figure S10. PPI networks among the proteins identified by Climp63- and DP1-IP.

Top 500 proteins with higher abundancies in both Climp63- and DP1-IPs from N2a cells were picked up, after which proteins enriched in DP1-IP or Climp63-IP were subtracted and analyzed using STRING database to identify the specific PPI network containing ER sheet or tubule proteins. A. The PPI network with sheet-enriched proteins contained several PPI clusters composed of the proteins related to Nuclear lumen, Ribonucleoprotein complex, Mitochondrion, and ER membrane. The sheet-enriched proteins, Climp63 and Canx, were found at center of the ER membrane cluster. VAPB and VAPA were also indicated. B. The PPI network with tubule-enriched proteins contained additional cluster composed of ER lumen proteins in addition to the above four clusters. In contrast, the components in nuclear lumen cluster were reduced. The tubule-enriched proteins, DP1 and Rtn3/4, were found at periphery of the ER membrane cluster.

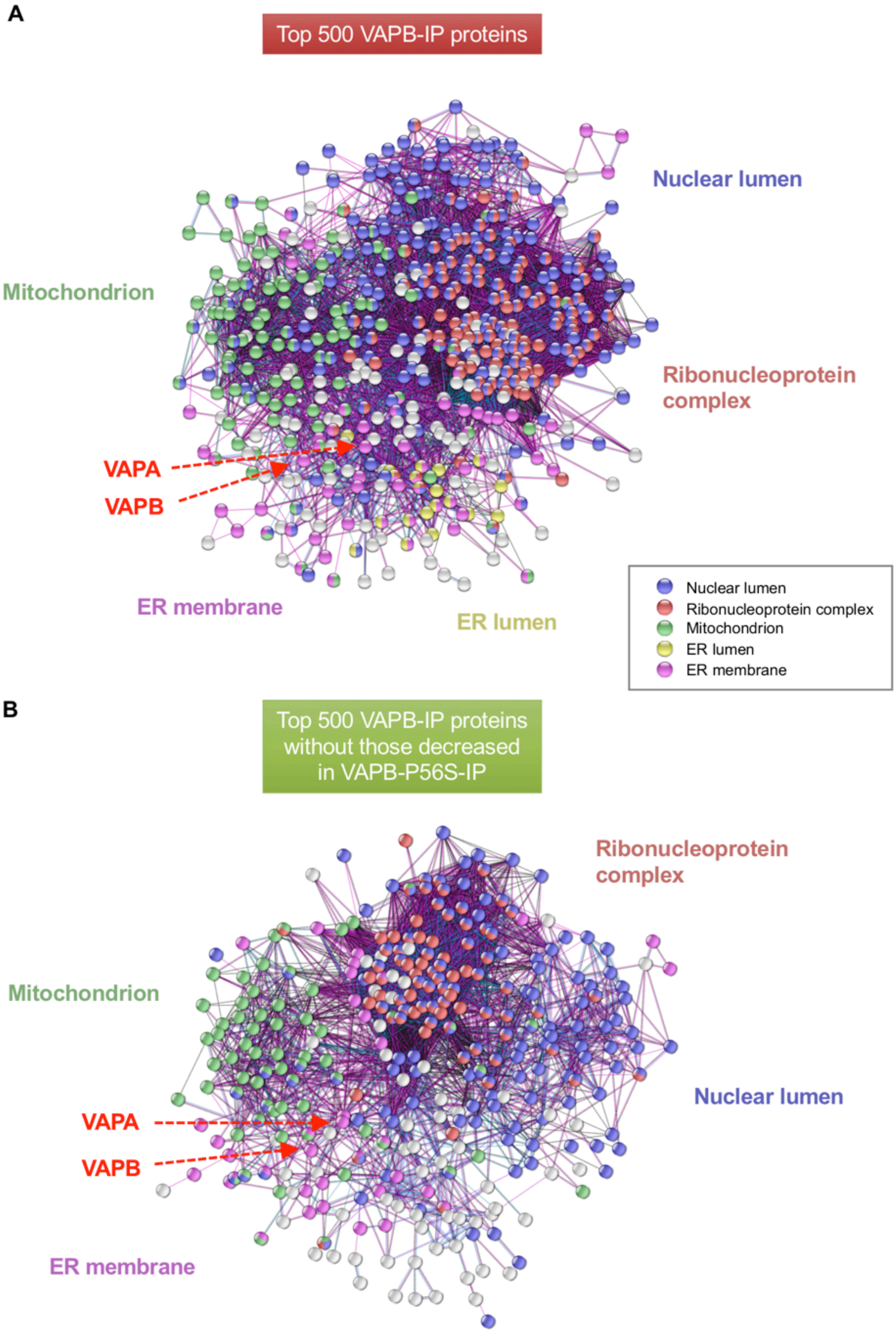
A
Top 500 VAPB-IP proteins
Nuclear lumen
Mitochondrion
Ribonucleoprotein complex
VAPA
VAPB
ER membrane
ER lumen
Nuclear lumen
Ribonucleoprotein complex
Mitochondrion
ER lumen
ER membrane
B
Top 500 VAPB-IP proteins without those decreased in VAPB-P56S-IP
Ribonucleoprotein complex
Mitochondrion
Nuclear lumen
VAPA
VAPB
ER membrane

Supplementary Figure S11. PPI networks among the proteins identified by VAPB-IP.

A. Top 500 proteins with higher abundancies in VAPB-wt-IP from N2a cells were analyzed using STRING database. The PPI network contained five clusters composed of proteins related to Nuclear lumen, Ribonucleoprotein complex, Mitochondrion, ER membrane and ER lumen. VAPB and VAPA were found at edge of the ER membrane cluster, close to the Mitochondrion cluster. B. The top 500 VAPB-wt-IP proteins without those decreased in VAPB-P56S-IP were analyzed using STRING database. Proteins in the Mitochondrion cluster were reduced, and a cluster of ER lumen proteins was unobserved.

Figure 2B

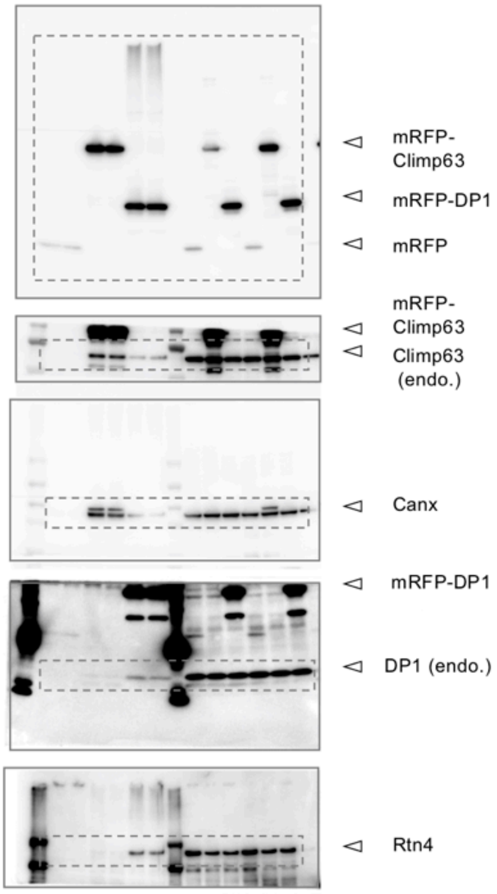

Figure S3A

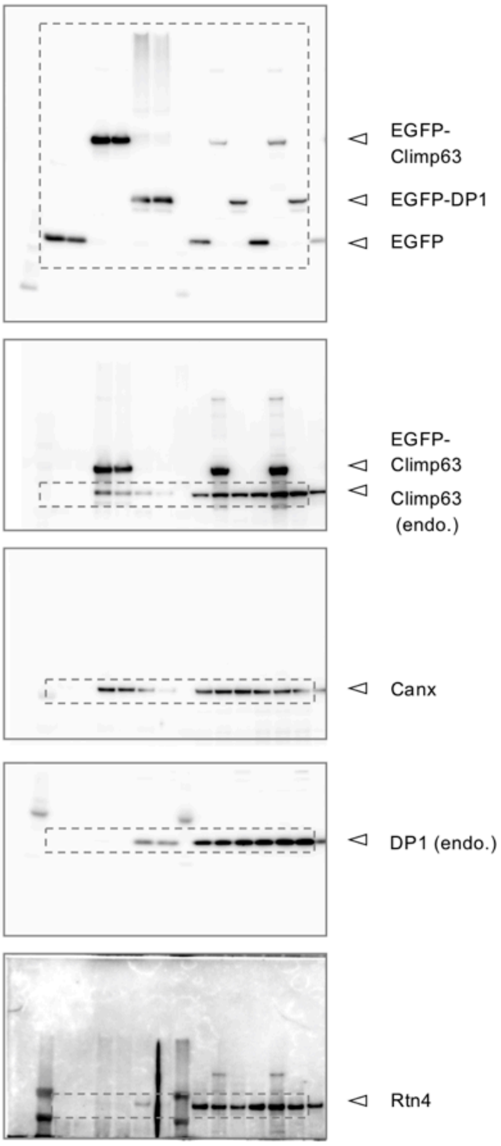

Figure S2A

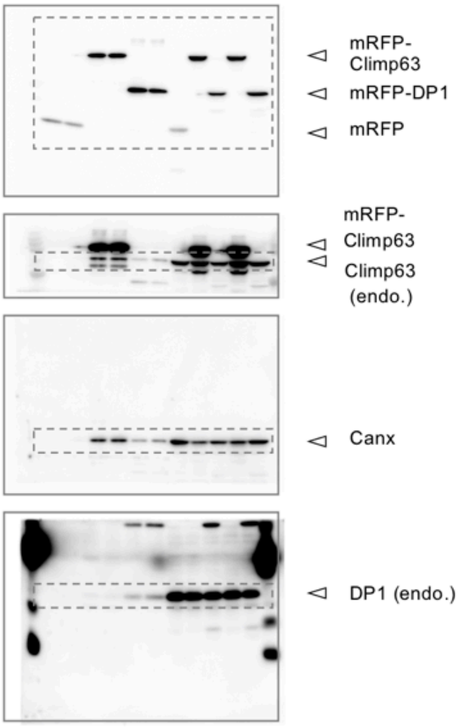

Figure 4A

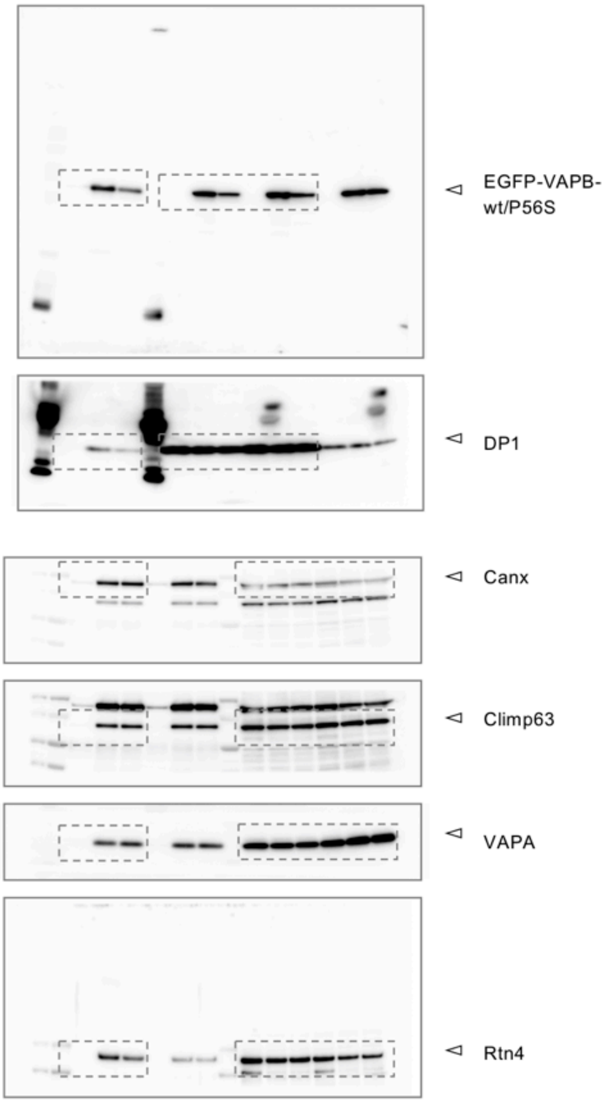

Figure 4C

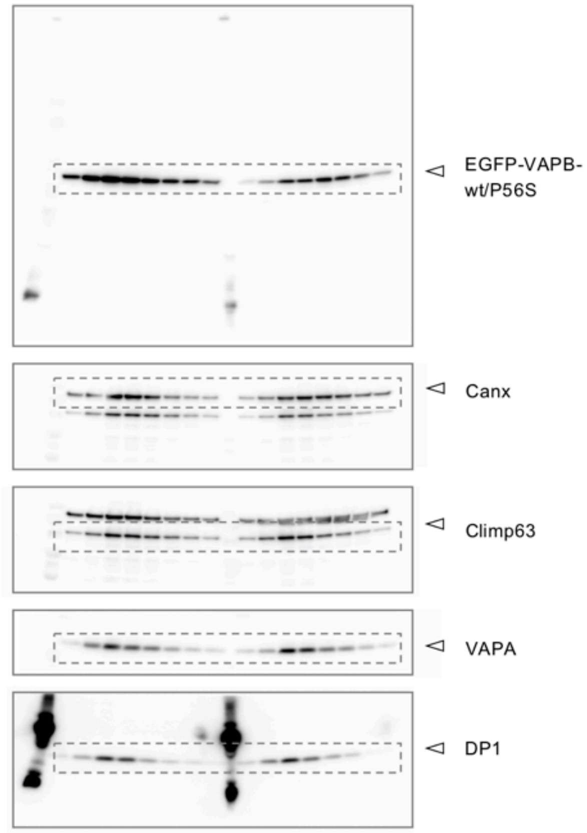

Figure S8A

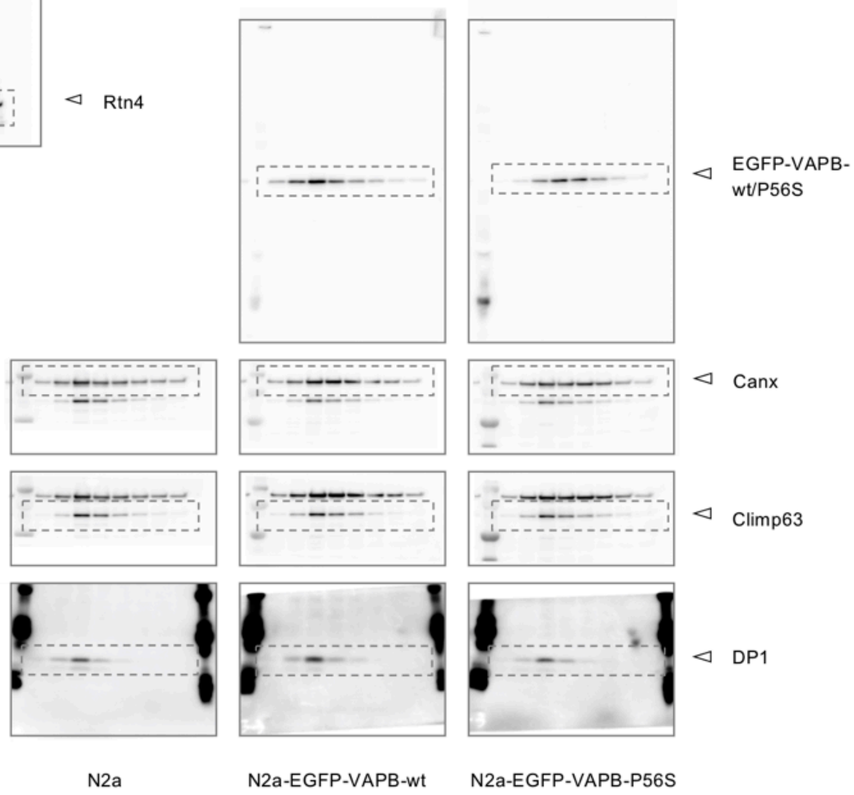

Supplementary Figure S12. Full-length images of Western blots.

Regions shown in the figures are enclosed by dotted lines. In some blots, we used transfer membranes cut horizontally. We also used mixed antibodies to detect two proteins (Canx and Climp63) simultaneously in some of the blots.
